# Inhibition of the IGF-1–PI3K–Akt–mTORC2 pathway in lipid rafts increases neuronal vulnerability in a genetic lysosomal glycosphingolipidosis

**DOI:** 10.1242/dmm.036590

**Published:** 2019-05-23

**Authors:** Tuba Sural-Fehr, Harinder Singh, Ludovico Cantuti-Catelvetri, Hongling Zhu, Michael S. Marshall, Rima Rebiai, Martin J. Jastrzebski, Maria I. Givogri, Mark M. Rasenick, Ernesto R. Bongarzone

**Affiliations:** 1 Department of Anatomy and Cell Biology, University of Illinois at Chicago, Chicago, IL 60612, USA; 2 Department of Physiology and Biophysics, University of Illinois at Chicago, Chicago, IL 60612, USA

**Keywords:** IGF-1, Neurodegeneration, Krabbe disease, Sphingolipid, Akt, mTOR, Membrane dysfunction, Neuroprotection, Lipid raft, Phosphorylation, GSK3β

## Abstract

Glycosphingolipid (GSL) accumulation is implicated in the neuropathology of several lysosomal conditions, such as Krabbe disease, and may also contribute to neuronal and glial dysfunction in adult-onset conditions such as Parkinson's disease, Alzheimer's disease and multiple sclerosis. GSLs accumulate in cellular membranes and disrupt their structure; however, how membrane disruption leads to cellular dysfunction remains unknown. Using authentic cellular and animal models for Krabbe disease, we provide a mechanism explaining the inactivation of lipid raft (LR)-associated IGF-1–PI3K–Akt–mTORC2, a pathway of crucial importance for neuronal function and survival. We show that psychosine, the GSL that accumulates in Krabbe disease, leads to a dose-dependent LR-mediated inhibition of this pathway by uncoupling IGF-1 receptor phosphorylation from downstream Akt activation. This occurs by interfering with the recruitment of PI3K and mTORC2 to LRs. Akt inhibition can be reversed by sustained IGF-1 stimulation, but only during a time window before psychosine accumulation reaches a threshold level. Our study shows a previously unknown connection between LR-dependent regulation of mTORC2 activity at the cell surface and a genetic neurodegenerative disease. Our results show that LR disruption by psychosine desensitizes cells to extracellular growth factors by inhibiting signal transmission from the plasma membrane to intracellular compartments. This mechanism serves also as a mechanistic model to understand how alterations of the membrane architecture by the progressive accumulation of lipids undermines cell function, with potential implications in other genetic sphingolipidoses and adult neurodegenerative conditions.

This article has an associated First Person interview with the first author of the paper.

## Abstract (precis)

**Summary:** The authors present a mechanistic model to understand how alterations of the membrane architecture by the progressive accumulation of sphingolipids undermines basic cell function, with potential implications for a variety of genetic sphingolipidoses and adult neurodegenerative conditions.

## INTRODUCTION

Glycosphingolipids (GSLs) are key components of neural membranes, and defects in their metabolism have negative consequences on neuronal and glial survival (Ballabio, 2009; Onyenwoke and Brenman, 2015; Settembre et al., 2008; Sural-Fehr and Bongarzone, 2016). Several lysosomal storage diseases (LSDs) are associated with defects in GSL metabolism and constitute excellent disease models to study the pathogenic mechanisms underlining GSL-driven neurodegeneration. Krabbe disease (KD) is an LSD caused by deficient activity of galactosyl-ceramidase (GALC) and the toxic accumulation of the GSL psychosine (galactosyl-sphingosine) (Igisu and Suzuki, 1984), and is compounded by irreversible neuronal and glial degeneration. Some of the downstream components of neuronal disease in KD include a dying-back axonopathy (Castelvetri et al., 2011), aberrant phosphorylation of cytoskeletal proteins (Cantuti-Castelvetri et al., 2012), dysregulation of axonal transport (Cantuti-Castelvetri et al., 2013), and motor and muscle deficits (Cantuti-Castelvetri et al., 2015). How GSLs such as psychosine elicit a spectrum of pathogenic mechanisms remains largely unclear.

Being a GSL, psychosine preferentially accumulates in, and disrupts the structure of, lipid rafts (LRs) (White et al., 2011, 2009). LRs are rigid platforms within the plasma membrane and relevant for the activation of multiple cellular signals such as insulin growth factor 1 (IGF-1) (Kavran et al., 2014), Akt (Arcaro et al., 2007) and others. Psychosine is known to downregulate Akt signaling *in vitro* and *in vivo* (Zaka et al., 2005); however, the exact mechanism of this inhibition is unknown. Akt is a direct downstream effector from the IGF-1 receptor (IGF-1R), is key for neuronal growth and survival, and is a crucial master kinase important in the regulation of the lysosomal-autophagosomal network. IGF-1 activates its receptor, IGF-1R, in LRs, followed by a recruitment of phosphoinositide 3-kinase (PI3K) to the activated receptor. Phosphatidylinositol (3,4,5)-trisphosphate [PtdIns(3,4,5)*P*_3_; PIP3] generation by PI3K is the key step in the recruitment of Akt to the plasma membrane, where it is phosphorylated by PDK1 and mTOR complex 2 (mTORC2), resulting in its activation. After release from the membrane, Akt regulates a myriad of downstream targets in the cytoplasm, including GSK3β and mTOR complex 1 (mTORC1) (Romanelli et al., 2009). In this context, we hypothesized that inhibition of the Akt pathway by psychosine involves the disruption of LR integrity and interferes with the proper activation of subsequent signaling within these domains.

Here, we show that Akt inhibition by psychosine specifically takes place within LR, where psychosine prevents the recruitment of the key upstream kinases PI3K and mTORC2 to these domains downstream of IGF-1R activation. This inhibition in Akt phosphorylation can be overcome by sustained stimulation with IGF-1 but only during a time window before psychosine accumulation reaches a threshold level. We also show a previously unknown aspect of mTORC2 activation that involves its recruitment to and phosphorylation in LRs, which may be relevant in other contexts such as neurodegenerative diseases and cancer.

## RESULTS

### Psychosine downregulates growth-factor-stimulated neuronal survival pathways

**Fig. 1.**
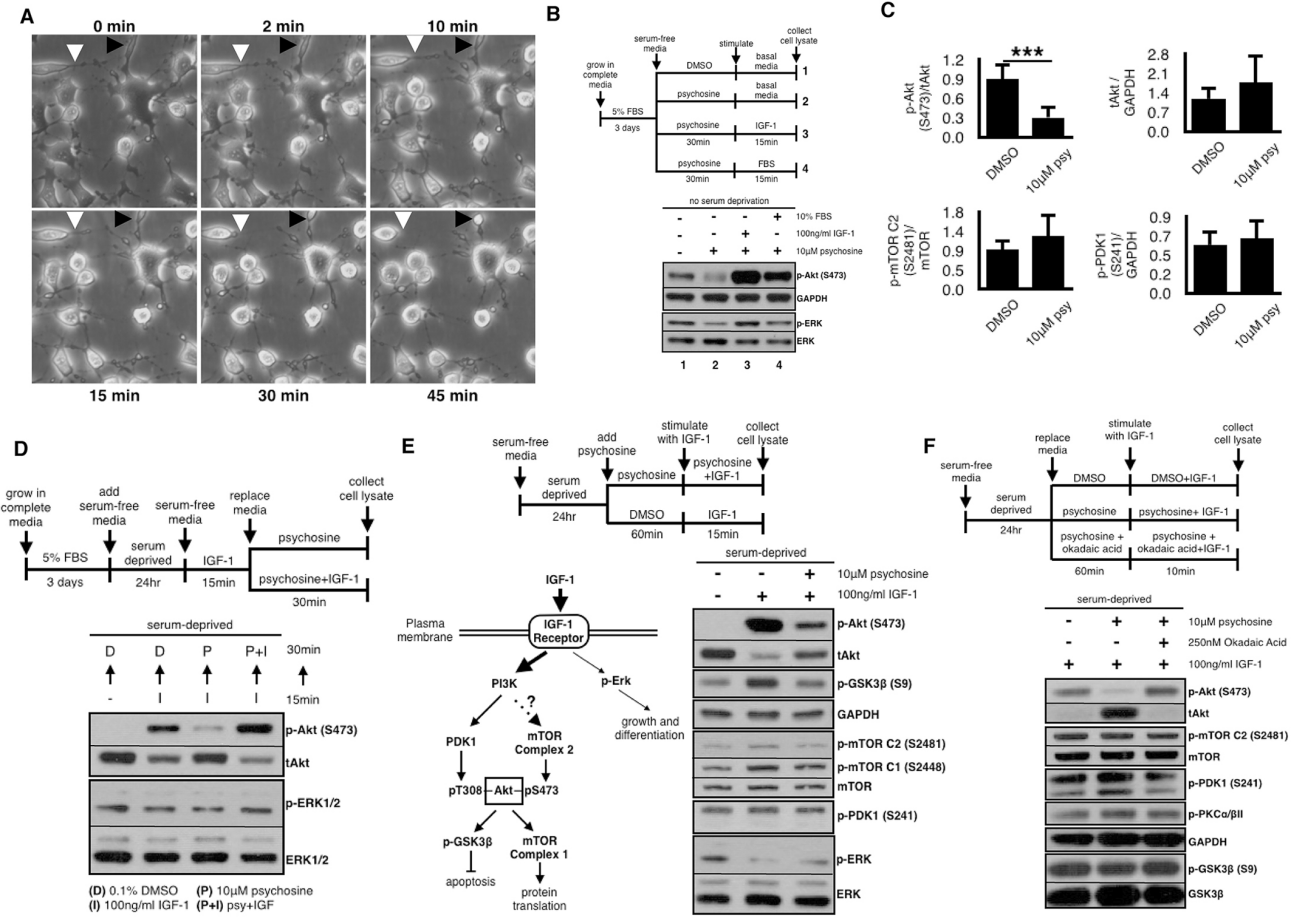
**Effects of psychosine on neuronal survival signaling in motor-neuron-like cells.** (A) Motor-neuron-like NSC34 cells were grown in complete culture media for 3 days at 37°C until ∼50% confluent. Cell media was replaced with serum-free media containing 10 μM psychosine and images from live cultures were taken at room temperature at the indicated time points. Psychosine causes visible changes in cellular morphology (i.e. loss of processes) without affecting cell viability under the conditions used in this study. White and black arrowheads each indicate a representative cell which retracts neurites over time. (B) NSC34 cells were grown in complete culture media until ∼50% confluent and treated for 30 min at 37°C with 10 μM psychosine in serum-free media (or 0.1% DMSO as vehicle). Cells were subsequently stimulated with the indicated growth factors (GFs) in the absence of psychosine. Whole-cell lysates were prepared and protein levels determined by immunoblotting. Image is a representative of at least three independent experiments with technical triplicates for each condition. Psychosine causes a rapid downregulation of existing cellular p-Akt and p-ERK levels, which can be overcome by GF stimulation. (C) Cells were grown in complete media until ∼60% confluent, and treated with 10 μM psychosine (or vehicle) in serum-free media for 30 min at 37°C. Phosphorylated and non-phosphorylated protein levels were determined by western blotting and quantified using the National Institutes of Health (NIH) ImageJ software. The data represent mean ratio±s.d. from three independent experiments with technical triplicates for each condition. ***P<0.0001. Inhibition of Akt phosphorylation is not due to a change in the phosphorylation levels of its direct upstream kinases, mTORC2 and PDK1. psy, psychosine; tAkt, total Akt. (D) Cells were grown in complete media until ∼50% confluent, followed by serum deprivation for 24 h to reduce protein phosphorylation to basal levels. Cells were stimulated with IGF-1 (100 ng/ml) for 15 min, followed by a 30 min treatment with either 10 μM psychosine alone, or 10 μM psychosine in combination with IGF-1 (100 ng/ml) at 37°C. Psychosine is unable to downregulate p-Akt levels under sustained IGF-1 stimulation. Image is a representative of at least three independent experiments. (E) Cells were serum deprived and pre-treated with 10 μM psychosine (or vehicle) for 1 h followed by either 15 min IGF-1 (100 ng/ml) alone, or in the continued presence of 10 μM psychosine at 37°C. Image is a representative of >10 independent experiments with technical triplicates for each condition. Prior exposure to psychosine significantly inhibits IGF-1-mediated Akt pathway activation. (F) Cells were pre-treated for 1 h with psychosine (10 μM) in the presence or absence of okadaic acid (250 nM), followed by IGF-1 (100 ng/ml) stimulation under continued presence of the same respective pre-treatment conditions at 37°C. Image is a representative of two independent experiments with technical triplicates for each condition. Psychosine-mediated loss in Akt phosphorylation can be rescued by broad inhibition of cellular protein phosphatases.

Motor neurons are particularly sensitive to the effects of psychosine (Castelvetri et al., 2011), and are a clearly affected cell population in children with Krabbe disease. Our study was carried out using the motor neuronal NSC34 cell line, a relevant *in vitro* model for psychosine toxicity studies (Castelvetri et al., 2011). NSC34 cells were grown in serum-free media containing 10 µM psychosine, resembling the level accumulated in the brain of sick twitcher mice (Spassieva and Bieberich, 2016). Cells that were incubated continuously at room temperature over a 70 min time period show changes in morphology evident as early as 10 min after psychosine addition: cells start retracting their processes and begin rounding up (Fig. 1A). Although all cells were still alive and attached by 45 min, most cells had lost their processes (Fig. 1A, white and black arrowheads).

The IGF-1–Akt pathway is known to be downregulated by psychosine (Cantuti-Castelvetri et al., 2015; Zaka et al., 2005; Teixeira et al., 2014), but the mechanism of this effect is unknown. To examine the dynamics of this regulation, we looked at the intracellular levels of phosphorylated Akt (p-Akt) and p-ERK in conditions where we detected a significant change in cell morphology (Fig. 1A, 30 min). Psychosine causes a rapid downregulation of both p-Akt and p-ERK by 30 min (Fig. 1B) despite no change in the total levels of the Akt upstream kinases PDK1 and mTORC2 (Fig. 1C). Downregulation in p-Akt is evident as early as 15 min after psychosine addition (Fig. S1A).

We observed that switching to growth media containing fetal bovine serum (FBS) following psychosine treatment rescued cell morphology (personal observation) and p-Akt levels (Fig. 1B). A similar effect was elicited by stimulating cells with IGF-1 (Fig. 1B). Although psychosine inhibits both p-Akt and p-ERK, subsequent FBS stimulation only rescues the phosphorylation of Akt and not that of ERK (Fig. 1B, compare lanes 2 and 4), suggesting that Akt is the main survival pathway regulated by growth factors (GFs) in NSC34 cells (Fig. S1D, compare lanes 1 and 2). Therefore, GF-stimulated Akt phosphorylation is sensitive to inhibition by psychosine in NSC34 cells within the timeframe tested.

### Psychosine inhibits IGF-1-mediated Akt activation

To better understand how psychosine leads to a reduction of p-Akt in neurons, we focused on the effect of psychosine specifically on IGF-1-mediated Akt activation. IGF-1 is essential for neuronal survival through the activation of Akt and ERK pathways, and it fully rescues p-Akt inhibition caused by psychosine (Fig. 1B). In addition, IGF-1 pathway components and their interactions are very well characterized (Fig. 1E). NSC34 cells were starved in serum-free media for 24 h to reduce GF-mediated cellular signaling to basal levels, followed by IGF-1 stimulation (100 ng/ml) for 15 min. The dynamics of IGF-1 pathway activation have not been previously studied in this cell line; therefore, we first determined which phosphorylation events are regulated by IGF-1 stimulation (Fig. S1B, lanes 1 and 2). IGF-1 leads to the phosphorylation of its receptor, followed by downstream phosphorylation of its known effectors: p-Akt (S473), p-GSK3β (S9) and p-p70S6K1 (T389). As control, p-PKCα/βII (Thr638/641), a GF-independent phosphorylation event, is not stimulated upon IGF-1 treatment in NSC34 cells. IGF-1 stimulates the phosphorylation of Akt at the expense of ERK, however (Fig. 1E, lanes 1 and 2), suggesting that IGF-1 preferentially activates p-Akt in NSC34 cells (Fig. 1D). Treatment with 10 µM psychosine in serum-free media for 30 min following IGF-1 stimulation causes a specific downregulation in p-Akt levels, but only in the absence of sustained IGF-1 stimulation (Fig. 1D). Similarly, psychosine is unable to downregulate p-Akt levels (and cause morphological changes) if psychosine incubation is done in complete media instead of serum-free media (data not shown), suggesting that continuous availability of GFs overrides psychosine inhibition of p-Akt.

We next investigated whether pre-treatment with psychosine prior to IGF-1 stimulation would inhibit initial pathway activation. Cells were serum deprived and treated with 10 µM psychosine for 1 h, followed by IGF-1 (100 ng/ml) for 15 min (Fig. 1E). Pre-treatment with psychosine significantly inhibits Akt pathway activation by IGF-1, despite its direct upstream kinases PDK1 and mTORC2 being intact (Fig. 1E,C). Inhibition of p-Akt as well as its downstream targets (p-GSK3β and p-p70S6K1) were dose dependent (Fig. S1B), cumulative over time (Fig. S1C) and independent of receptor phosphorylation (Fig. S1B, compare lanes 2 and 6). We noticed that IGF-1 stimulation also triggered morphological changes in cells, such as inducing neurite-like formations. Similarly to as shown in Fig. 1A, psychosine also inhibited these IGF-1-induced formations in a dose-dependent manner that correlated with the level of p-Akt inhibition (data not shown). Neurite extension in NSC34 cells depends on PI3K, upstream of Akt, and therefore implies that IGF-1 may regulate neurite extension via PI3K-dependent Akt activation in our culture. Surprisingly, inhibition of p-Akt is achieved only when psychosine is continuously kept in the media throughout the IGF-1 stimulation period (Fig. S1F). Akt phosphorylation is fully recovered if psychosine is subsequently removed from the media during IGF-1 treatment, except when using higher doses of psychosine (20-30 µM; data not shown). Therefore, psychosine levels and the duration of exposure appear to be important factors in determining whether pathway inhibition can be overcome by GFs *in vitro*. Overall, these results suggest that damage to neuronal survival pathways begins shortly after cellular exposure to psychosine, accumulates over time, and is independent of cellular proliferation state. Most importantly, there may only be a brief time window during which inhibitory effects are reversible before psychosine accumulates to levels at which it causes irreversible neuronal damage.

### Cellular phosphatases in psychosine-mediated inhibition of Akt phosphorylation

Psychosine causes an upregulation in the activities of cellular protein phosphatases PP1 and PP2A, leading to a loss in the phosphorylation of GSK3β and neurofilaments (Cantuti-Castelvetri et al., 2013, 2012). Therefore, we asked whether an increase in cellular phosphatase activities would be responsible for the observed p-Akt (S473) loss, especially when its direct upstream kinase, mTORC2, is intact and functional (mTORC2 activity marker p-S2481 and a downstream target, p-PKCα/βII-Thr638/641, remain unchanged; Fig. S1B). Cells were serum deprived for 24 h, pre-treated for 1 h with 10 µM psychosine with or without 250 nM okadaic acid (OA), followed by IGF-1 (100 ng/ml) stimulation for 10 min (Fig. 1F). Although OA completely rescued psychosine-mediated inhibition of Akt phosphorylation following IGF-1 stimulation (Fig. 1F), it had an opposite effect on p-Akt under basal conditions in the absence of IGF-1 stimulation (Fig. S1E). Additionally, specific inhibition of protein phosphatases PP1 and PP2A (by 1 µM tautomycin and 200 nM fostriecin, respectively), or the inhibition of lipid phosphatases PTEN and Ship2 (by 200 nM SF1670 and 10 µM Ship2 inhibitor II, respectively), all had similar effects in rescuing p-Akt, p-p70S6K1 and p-ERK (Fig. S1G). Protein/lipid phosphatases are the main mechanism employed by cells to quench and fine-tune signal intensity following pathway activation, and the above phosphatases have all been implicated as negative regulators of the IGF-1R pathway. It is perhaps not surprising that inhibition of any of these upstream negative regulators would lead to an increase in pathway activity and hence rescue Akt phosphorylation. Therefore, we conclude that, even though a psychosine-mediated increase in phosphatase activity may be contributing to reduced Akt phosphorylation, it does not appear to be the sole mechanism of inhibition by psychosine.

### Psychosine modulates PI3K-dependent Akt pathway activation downstream of IGF-1R phosphorylation

**Fig. 2.**
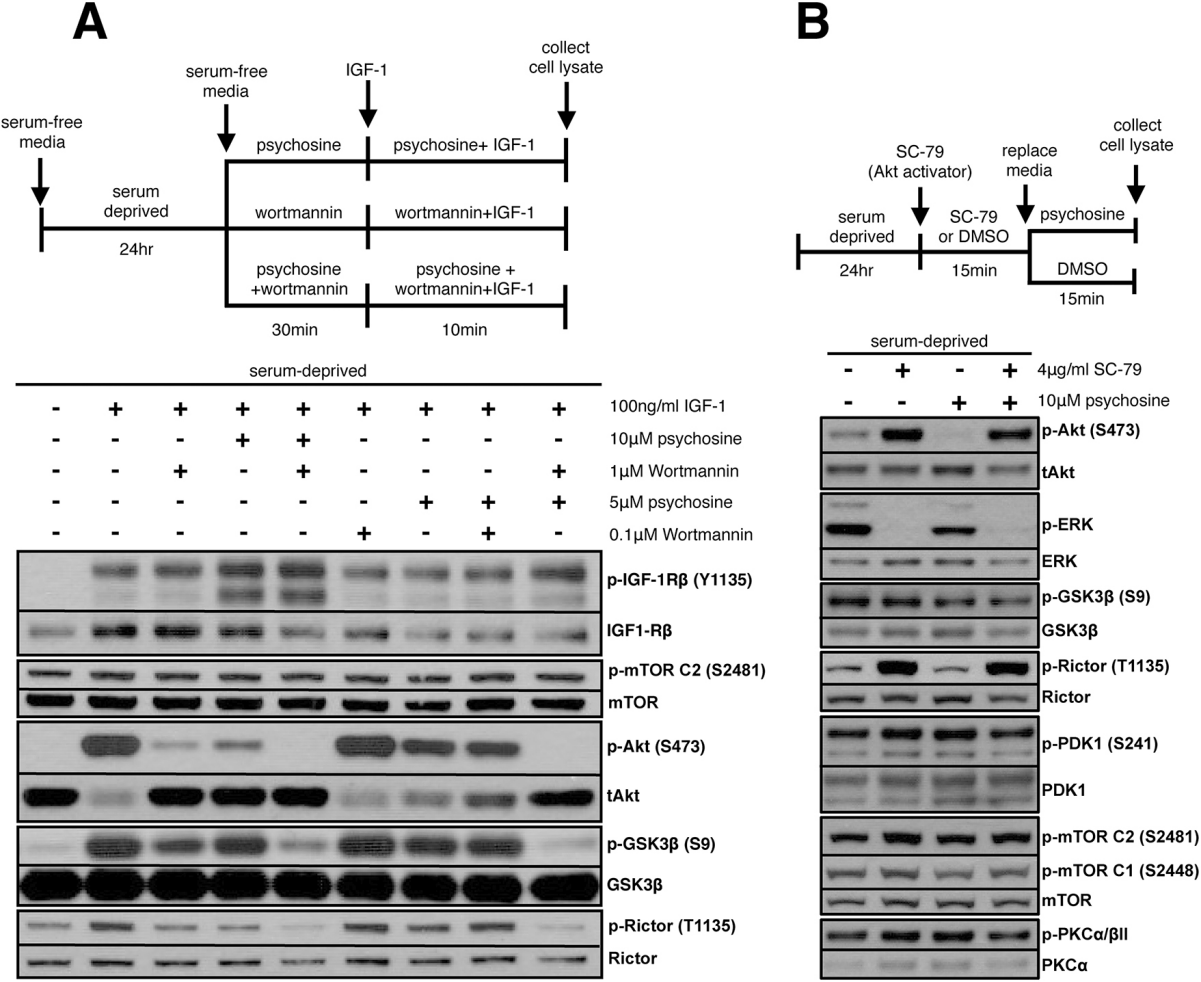
**Psychosine inhibits PI3K-dependent Akt phosphorylation downstream of IGF-1R activation at the membrane.** (A) Cells were serum deprived and pre-treated for 30 min with either psychosine (5 μM, 10 μM), the PI3K/p110α inhibitor wortmannin (0.1 μM, 1 μM) or a combination of the two. Cells were subsequently stimulated with IGF-1 (100 ng/ml) under the same respective pre-treatment conditions. Image is a representative of five independent experiments with technical triplicates for each condition. Psychosine inhibition of the Akt pathway is independent of IGF-1R activation and is synergistic with PI3K inhibition. (B) Cells were serum deprived and pre-treated for 15 min with a PIP3 mimetic, SC-79, followed by 10 μM psychosine for another 15 min. Image is a representative of at least three independent experiments with technical triplicates for each condition. SC-79 stimulates Akt phosphorylation within the cytoplasm, bypassing the membrane activation step. When activated within the cytosol, p-Akt levels appear resistant to downregulation following subsequent psychosine treatment. SC-79 also stimulates mTORC2 auto-phosphorylation and Rictor phosphorylation.

PIP3 generation by PI3K in response to receptor activation is the main mechanism for recruiting Akt to the plasma membrane, where it is phosphorylated by the upstream kinases PDK1 and mTORC2. In our NSC34 culture system, Akt inhibition by psychosine is independent from and downstream of IGF-1R activation (Fig. S1B, compare lanes 2 and 6), and can be antagonized by elevating PIP3 levels indirectly by inhibiting the lipid phosphatases PTEN and Ship2 (Fig. S1G). Therefore, we hypothesized that there would be synergy between the inhibition of PI3K activity, and hence levels of PIP3 generated, and the inhibition of p-Akt by psychosine, especially when combined at suboptimal doses. Cells were serum deprived and pre-treated for 30 min with psychosine (5 µM, 10 µM), PI3K/p110α inhibitor wortmannin (0.1 µM, 1 µM), or a combination of both at each dose. Cells were subsequently stimulated with IGF-1 (100 ng/ml) under the same respective pre-treatment conditions and the levels of p-Akt and its downstream target p-GSK3β were assessed in whole-cell lysates using western blotting. As expected, either 10 μM psychosine or robust inhibition of PI3K activity by 1 µM wortmannin significantly but not completely blocks Akt phosphorylation downstream of receptor activation (Fig. 2A). Similarly, suboptimal doses of psychosine (5 µM) or wortmannin (0.1 µM) each cause partial inhibition. However, a combination of psychosine and wortmannin completely eliminates any remaining Akt phosphorylation even at suboptimal doses, resulting in a complete loss of GSK3β phosphorylation. IGF-1R phosphorylation is intact despite the loss in downstream signaling, implying inhibition by psychosine takes place downstream of receptor activation. Phosphorylation of the receptor may even be increased when cells are treated with 10 µM psychosine, and we have observed this repeatedly, although we have not investigated this aspect in detail (Fig. S1B,F). IGF-1R is unique in that it is embedded in the plasma membrane as an inactive hetero-tetramer, and this autoinhibition is released upon ligand binding as opposed to the activation of receptors such as EGFR and insulin receptor, for which dimerization through ligand binding is required for activation. Additionally, plasma membrane rigidity does not affect ligand binding to IGF-1R (Yorek et al., 1989), and rafts/caveolae are required only for downstream signaling and not receptor activation by the ligand (Huo et al., 2003). This could explain why receptor phosphorylation is not affected by psychosine even in the presence of presumed rigidification of the plasma membrane and disruption of LRs. Thus, psychosine treatment is synergistic with PI3K catalytic inhibition, suggesting that psychosine modulates PI3K activity and/or PIP3 levels, leading to a loss in Akt pathway activation independent of receptor phosphorylation and without compromising the integrity of the Akt upstream kinases mTORC2 or PDK1.

### Psychosine inhibits PI3K-stimulated mTORC1/p70S6K1 activities

PI3K is a key mediator of GF-stimulated activities within cells. It regulates cellular survival/proliferation by activating Akt/ERK pathways, which in turn leads to cellular growth/protein translation through the stimulation of mTORC1/p70S6K1 activities. We hypothesized that, if psychosine is modulating PI3K activity towards the Akt pathway, as evidenced from its synergism with wortmannin (Fig. 2A), the effects may also extend to an inhibition of mTORC1/p70S6K1 pathways. Phosphorylation of p70S6K1 at T389 is an established readout of GF-stimulated mTORC1 activity, and phosphorylation of Rictor at T1135 is one readout for GF-stimulated p70S6K1 activity. We chose to look at p-Rictor since this modification is an indirect marker for PI3K-dependent mTORC2 activity in IGF-1-stimulated cells, and correlates with p-mTOR (S2481) and p-Akt (S473) levels. Neither p70S6K1-mediated Rictor phosphorylation at T1135 nor mTORC1-mediated p70S6K1 phosphorylation at T389 has previously been studied in NSC34 cells. Therefore, we first sought to establish whether p-Rictor and p-p70S6K1 were appropriate readouts for p70S6K1 and mTORC1 activities, respectively.

Treatment of cells with psychosine inhibits IGF-1-stimulated p70S6K1 phosphorylation in a dose-dependent manner (Fig. S1B). Similarly, IGF-1 stimulates, and 1 µM wortmannin significantly inhibits, p70S6K1 phosphorylation (i.e. mTORC1 activity) and its downstream target, p-Rictor (T1135) (i.e. p70S6K1 activity) (Fig. 2A and Fig. S2A). We therefore conclude that both are activated by the IGF-1R–PI3K axis in NSC34 cells. Treating cells with 10 µM psychosine in addition to 1 µM wortmannin eliminates p70S6K1 phosphorylation (Fig. S2A), which can be rescued by elevating PIP3 levels through PTEN/Ship2 inhibition (Fig. S1G). p-Rictor (T1135) follows the same pattern (Fig. 2A and data not shown). The phosphorylation of p70S6K1 and Rictor mirror that of Akt, suggesting that both are regulated by p-Akt. Therefore, inhibition by psychosine extends to mTORC1 and p70S6K1 activities downstream of Akt and beyond p-GSK3β. Our data suggest that psychosine negatively regulates PI3K activity in response to GF stimulation. The resulting effect is the inhibition of Akt phosphorylation and subsequent deregulation of its targets, including abnormal activation of GSK3β and inhibition of mTORC1/p70S6K1 pathways.

### Cytosolic activation of Akt is resistant to inhibition by psychosine

Psychosine accumulates at the plasma membrane, where PIP3 is also generated by PI3K, and our data suggest that they may antagonize each other to regulate Akt phosphorylation. We therefore hypothesized that inhibition of Akt phosphorylation by psychosine may be confined to the plasma membrane compartment, in which case we would be able to bypass this inhibition by uncoupling Akt activation from its pre-requisite recruitment to the membrane. The small molecule SC-79 is a soluble PIP3 mimetic that leads to Akt activation by directly binding to its pleckstrin homology (PH) domain, and rendering the protein in a conformation amenable for phosphorylation by upstream kinases in the absence of GF stimulation (Jo et al., 2012). Therefore, one can bypass the PIP3-dependent membrane recruitment step and instead stimulate Akt within the cytoplasm using SC-79. Serum-starved NSC34 cells were stimulated with SC-79 (4 µg/ml) for 15 min, followed by psychosine (10 µM) treatment for another 15 min, and whole-cell lysates were analyzed for protein phosphorylation using western blots (Fig. 2B). SC-79 treatment led to a strong stimulation of Akt phosphorylation along with a complete inhibition of ERK phosphorylation. Although we have not investigated it further, ERK inhibition is likely mediated by Raf in response to cytosolic Akt activation under these specific conditions. Surprisingly, the increase in p-Akt levels did not result in an increase in the phosphorylation of its direct target, GSK3β, as we see in IGF-1 stimulation. Instead, SC-79 stimulated the phosphorylation of p70S6K1 and its target Rictor (Fig. 2B, Fig. S2C), suggesting distinct regulation of Akt targets based on the compartment in which Akt is activated (Adam et al., 2007). As expected, psychosine treatment decreased basal p-Akt levels; however, it was unable to do so following stimulation with SC-79. This suggests that, once activated within the cytoplasm, cytosolic Akt is not subject to inhibition by psychosine (Fig. 2B). This result, along with the observation that inhibition by psychosine can be enhanced by decreasing PI3K catalytic activity at the membrane (Fig. 2A), points at a membrane-dependent step in the activation cascade as being the most sensitive to psychosine. Akt phosphorylation is largely unaffected by psychosine if it is initiated within the cytoplasm.

### Psychosine impairs Akt phosphorylation by inhibiting mTORC2 activation at the cell membrane

Upon GF stimulation, Akt protein first translocates to the plasma membrane for activation by upstream kinases PDK1 and mTORC2, then shuttles back to the cytoplasm and the nucleus to phosphorylate its downstream targets, thereby creating different pools of active Akt protein based on its localization (membrane-associated Akt versus cytosolic versus nuclear Akt). Results from our studies using wortmannin and SC-79 suggest that a membrane-associated step other than receptor phosphorylation in the Akt activation cascade may be compromised by psychosine. To further understand the mechanism of this inhibition, we compared the dynamics of IGF-1-mediated pathway activation in the presence or absence of psychosine within distinct subcellular compartments. Since this has not been studied in NSC34 cells before, we first determined the subcellular localization of IGF-1R pathway components and their phosphorylation in response to IGF-1 stimulation (Fig. S3A). Cells were serum-deprived for 6 h followed by IGF-1 (100 ng/ml) stimulation for 15 min, and were subjected to fractionation of subcellular compartments. IGF-1 leads to IGF1-Rβ and Akt phosphorylation at the membrane, with subsequent translocation of p-Akt to the cytoplasm. p70S6K1, its target Rictor, as well as mTORC2 are also phosphorylated at the membrane in response to IGF-1 pathway activation, but not ERK1/2 or PKCα. Membrane-associated p70S6K1 likely catalyzes Rictor phosphorylation within that compartment, since an exclusively cytoplasmic target of p70S6K1, p-IRS-1 (S636/639), is phosphorylated only within the cytoplasmic fraction (Ozes et al., 2001). Similarly, membrane-associated Akt phosphorylation is likely the result of mTORC2 activation (autophosphorylation at S2481) specifically at the membrane. In summary, IGF-1 treatment of NSC34 cells leads to the stimulation of mTORC2, Akt, p70S6K1 and Rictor phosphorylation, presumably in that same order, at the cell membrane.

**Fig. 3.**
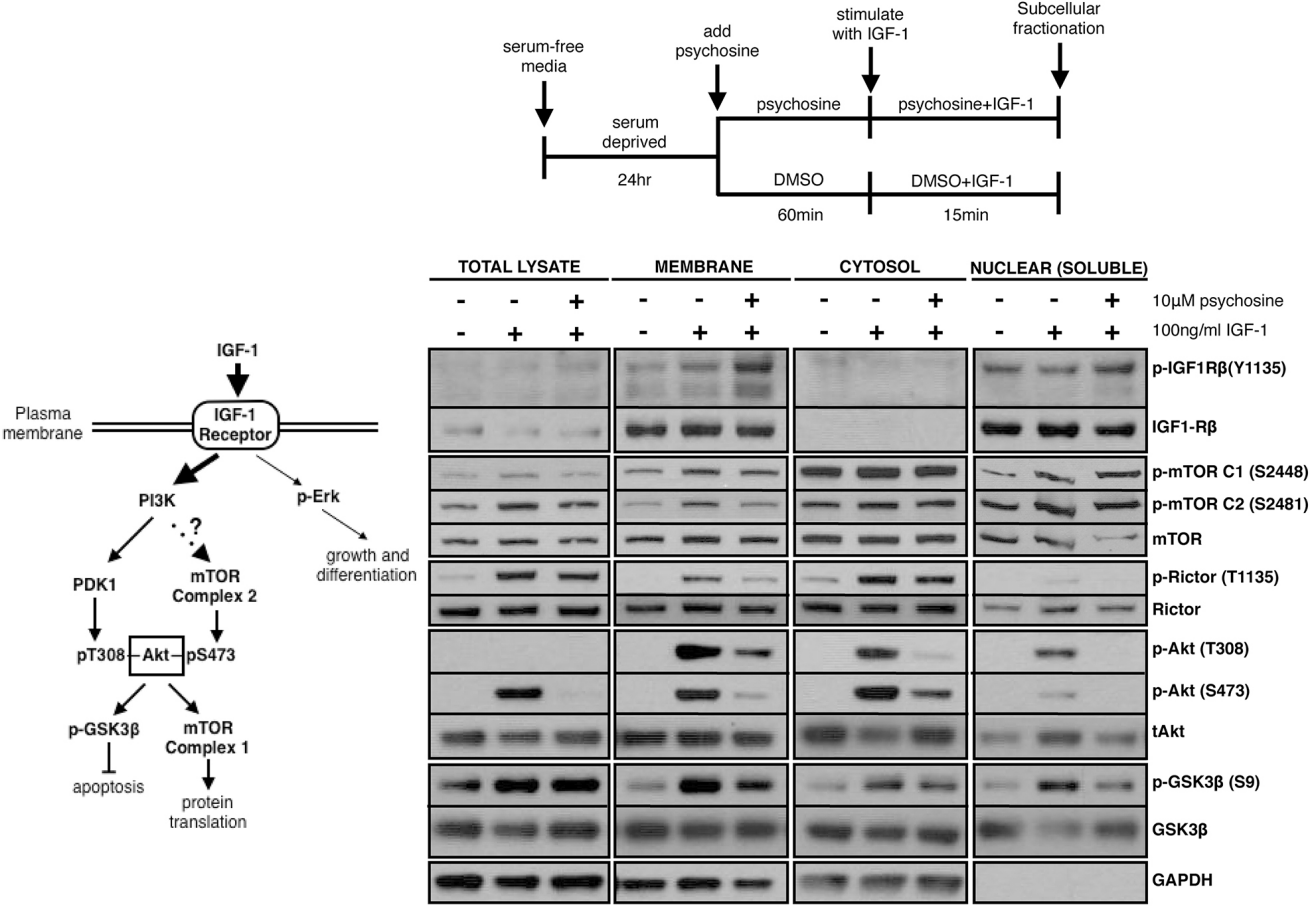
**Psychosine impairs Akt phosphorylation by inhibiting mTORC2 activation specifically at the cell membrane.** Left: components of the IGF-1R pathway analyzed in this study. p-IRS-1 (S636/639) is a surrogate for mTORC1 activity in the cytoplasm, whereas components including and upstream of p-p70S6K are found both on the membrane and in the cytoplasm (see Fig. S3C). PKCα is a target of mTORC2 that does not respond to IGF-1 stimulation in NSC34 cells. All other components are regulated by IGF-1. Serum-deprived NSC34 cells were incubated with 10 μM psychosine for 1 h, followed by IGF-1 (100 ng/ml) stimulation for 15 min in the continued presence of psychosine. At the end of stimulation, cytosolic, membrane-associated and nuclear proteins (soluble, non-chromatin bound) were isolated by subcellular fractionation, and levels of phospho-Akt and other pathway components were analyzed by immunoblotting. Psychosine inhibits mTORC2 activation (i.e. phosphorylation) and subsequent Akt phosphorylation specifically at the cell membrane. The loss in Akt phosphorylation is further mirrored in downstream subcellular compartments. The figure was assembled from data from a single experiment, and is a representative image of at least three independent experiments.

Next, we pre-treated cells with 10 µM psychosine for 1 h, followed by IGF-1 (100 ng/ml) stimulation with continued presence of psychosine. At the end of stimulation, we subjected cells to subcellular fractionation and compared phosphorylation levels in each fraction with total lysates (Fig. 3, Fig. S3C). As expected, psychosine treatment caused a significant inhibition of Akt activation in whole lysates without inhibiting receptor phosphorylation. This Akt inhibition is also reflected separately within the membrane, cytoplasmic and nuclear fractions. To our surprise, psychosine also caused an inhibition of mTORC2 phosphorylation, but not that of mTORC1, specifically at the membrane, an effect likely missed in total lysates due to dilution by the large volume of the cytoplasmic fraction. Based on this observation, we predict that the decrease in p-Akt levels in the cytoplasm and nucleus are collateral to an initial absence of Akt phosphorylation at the plasma membrane due to failed mTORC2 activation. Furthermore, our data suggest that Akt phosphorylation within the cytoplasmic and nuclear compartments can be rescued by IGF-1 stimulation at the plasma membrane when psychosine is present (Fig. S3D, Fig. 1D).

Another consideration for the inhibition of mTORC2 by psychosine is a corresponding inhibition of Rictor phosphorylation, an mTORC2-specific component and an indirect activity marker (Figs 2A and 3). Rictor phosphorylation by p70S6K1 regulates the activity of intact mTORC2 towards Akt (Dibble et al., 2009). In our system, there is a positive correlation between the stimulation of PI3K/p-Akt and the levels of p-Rictor (Figs 2 and 3, Fig. S3A,B) at least within the timeframe that we have tested. Stimulation of Rictor phosphorylation is mediated by mTORC1/p70S6K1 downstream of Akt, as the mTOR inhibitor rapamycin inhibits SC-79-stimulated Rictor phosphorylation (Fig. S2C). We should also note that there is appreciable Rictor phosphorylation in the absence of p-Akt (Fig. 3, see total lysate and cytosolic fraction), and even in the absence of p70S6K1 activation under basal conditions (Fig. S2C, first lane). This suggests that Rictor is under the regulation of different kinases under different conditions, with p70S6K1 being the main kinase under IGF-1 stimulation. We have not investigated the contribution of p-Rictor to overall mTORC2 activity in detail; however, we expect that the decrease in p-Rictor levels might additionally contribute to mTORC2 inhibition by psychosine, especially since Rictor translocation out of the nucleus is regulated by phosphorylation (Rosner and Hengstschlager, 2008).

### Psychosine alters the recruitment of IGF-1R pathway components to membrane lipid rafts

Psychosine is a positively charged lipid with strong affinity to LRs (White et al., 2011, 2009), where it disrupts PKC signaling (White et al., 2009; Yamada et al., 1996). LR integrity is essential for the activation of the IGF-1R pathway and the phosphorylation of Akt. LR disruption due to psychosine accumulation has previously been shown to dysregulate neurotrophic TrkA-receptor recruitment to LRs and a failure to activate downstream Akt/ERK pathways in *in vitro* differentiated twitcher dorsal root ganglia (Teixeira et al., 2014). We asked whether defective recruitment of IGF-1R, or its downstream components, to LRs would also be the cause of downstream signaling defects in our motor neuron culture.

**Fig. 4.**
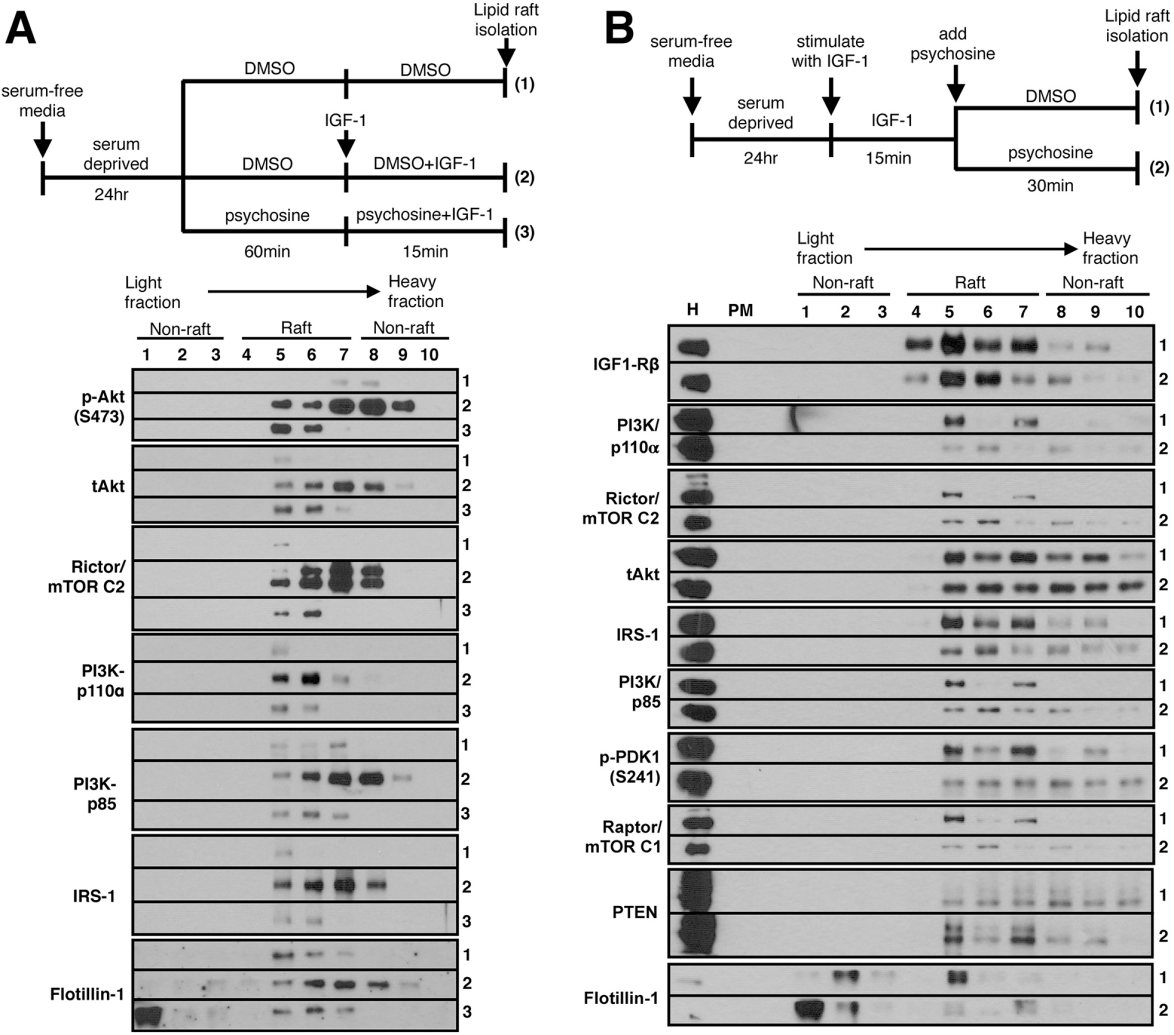
**Psychosine alters the recruitment of IGF-1R pathway components to membrane lipid rafts (LRs).** (A) Cells were serum deprived and pre-incubated with 10 μM psychosine for 1 h followed by IGF-1 (100 ng/ml) stimulation for 15 min in the continued presence of psychosine. At the end of treatment, cells were homogenized (H) and plasma membranes (PM) were isolated from post-nuclear homogenates based on their density difference on a Percoll gradient. Purified plasma membrane fractions were sonicated to release LRs and further subjected to fractionation on a 5-35% sucrose gradient. Fractions 1-10 (low to high density) were collected, and proteins in each fraction were precipitated and resuspended in an equal volume of loading buffer. Equal volumes of isolated fractions were loaded in each lane and subjected to immunoblotting. The figure is a representative image from three independent experiments. A lack of Akt activation in response to IGF-1 stimulation is due to a failure to recruit upstream kinases to LR domains when psychosine is present (compare to Fig. 1). (B) Cells were serum deprived and stimulated with IGF-1 (100 ng/ml) for 15 min, followed by 10 μM psychosine for an additional 30 min in the absence of IGF-1. The figure is a representative image from two independent experiments. Psychosine causes the movement of the LR marker flotillin-1 into low-density fractions, as well as a redistribution of pathway components out of LRs.

NSC34 cells were serum deprived and pre-treated with 10 µM psychosine for 1 h, followed by IGF-1 (100 ng/ml) stimulation for 15 min with continued presence of 10 µM psychosine. Cells were homogenized (H) and post-nuclear plasma membrane (PM) fractions were isolated using 30% Percoll. Purified PM was sonicated and subjected to fractionation on a 5-35% sucrose gradient overnight using a detergent-free method. Fractions 1-10 (low to high density) were collected, and proteins in each fraction were precipitated and resuspended in an equal volume of loading buffer. An equal volume of resuspended protein from each fraction was loaded in individual lanes and subjected to immunoblotting. Under basal conditions, there is minimal Akt protein at the PM (Fig. 4A, row 1). Upon IGF-1 stimulation, there is robust Akt phosphorylation both within and outside of LRs (Fig. 4A, row 2). However, after pre-treatment with psychosine, there is limited Akt phosphorylation upon IGF-1 stimulation, and it is restricted to LR fractions (Fig. 4A, row 3). Low-level Akt activation in the presence of psychosine is consistent with our previous observations (Fig. 1); however it is surprising to see this pool restricted only to LRs in the presence of psychosine. Similarly, we see a lack of recruitment of upstream pathway components, including IRS-1, PI3K and Rictor (mTORC2), to LRs (Fig. 4A). This suggests that the failure in the activation of Akt is the result of a failure to recruit its upstream regulators PI3K and mTORC2 to LR compartments in the presence of psychosine. This is likely due to a disruption in the LR structure, as the LR marker flotillin-1 is markedly shifted out of rafts into the lowest-density fraction. Therefore, activation of mTORC2 by autophosphorylation in response to IGF-1 may require its recruitment initially to intact LRs from non-raft regions. This could also explain the specific inhibition of mTORC2 phosphorylation at the membrane in our subcellular fractionation experiments (Fig. 3).

We next asked whether we would see a similar defect in LR recruitment in cells that have first been stimulated with IGF-1 followed by psychosine treatment, as opposed to pre-treatment with psychosine followed by IGF-1. Although the outcome is analogous in terms of decreased Akt phosphorylation overall (compare Fig. 1D versus 1E), the underlying assumption is different. In the former situation, one would be assessing the initial activation of the pathway after cells have been exposed to psychosine for a period of time, whereas the latter situation would be assessing the inhibition of an already active pathway. NSC34 cells were serum deprived and stimulated with IGF-1 (100 ng/ml) for 15 min, followed by 10 µM psychosine for an additional 30 min in the absence of IGF-1 (Fig. 4B). LR fractions were isolated as in Fig. 4A, and equal amounts of each fraction were analyzed using western blotting. Under IGF-1-stimulated conditions, IGF1-Rβ, PI3K, PDK1, Rictor (mTORC2) and Raptor (mTORC1) are all restricted to LR fractions. Akt, however, is distributed equally between raft and non-raft regions. Psychosine treatment causes a redistribution of pathway components between raft and non-raft fractions. In psychosine-treated cells, we see an increase in Rictor/mTORC2, IRS-1, PI3K/p85, PI3K/p110α, p-PDK1 and Raptor/mTORC1 in non-raft fractions, whereas PTEN is decreased. There may also be a slight increase in Akt levels in non-raft fractions, although the overall distribution pattern looks similar.

In addition, the level of IGF-1R is not affected, consistent with our previous observation that inhibition is downstream of receptor activation (Figs S1B, S2A). Similar to conditions in Fig. 4A, flotillin-1 was also redistributed out of LRs into low-density fractions, suggesting that LRs are disrupted as early as 30 min after psychosine addition. This disruption is likely the cause of the movement of downstream components out of LRs.

Akt distribution was not significantly affected upon psychosine treatment especially when we did not detect any Akt phosphorylation under this condition (data not shown). This is in contrast to our observation in Fig. 4A, where there is a lack of Akt recruitment to LRs upon IGF-1 stimulation after cells have been exposed to psychosine for a period of time. We predict that the lack of Akt phosphorylation in Fig. 4B is not due to a lack of Akt recruitment to the plasma membrane, but instead due to an exclusion of upstream kinases from LRs. The difference may be due to the dynamics associated with psychosine-mediated structural perturbations in LRs when pathway components are already residing there versus when they are initially recruited. Therefore, judging based solely on the final levels of cellular p-Akt in various conditions that we have tested may not be an accurate measure of Akt activity per se, especially in whole-cell lysates, as it may not be reflective of the Akt activation pattern at the membrane/LR level. This is important because Akt can phosphorylate different targets depending on whether it is activated within or outside of LRs, and therefore this would have implications for therapy. Indeed, if cells are pre-treated with 10 µM psychosine, and stimulated by IGF-1 (100 ng/ml) after psychosine has been removed (as opposed to continually present), we see yet another picture for LR distribution (Fig. S4). In this scenario, we can fully rescue the levels of Akt phosphorylation judging from whole-cell lysates (Fig. S1F, compare lanes 2 and 4); however, the pattern of p-Akt distribution on the plasma membrane is altered (Fig. S4). There is ectopic recruitment and activation of Akt outside of LRs in cells treated with psychosine prior to IGF-1 stimulation, but only when psychosine is removed from media during IGF-1 incubation. This appears to result from an increased recruitment of PI3K components p110α/p85, followed by PDK1 and mTORC2, to non-raft regions (compare fractions 8-10 between samples 2 and 3 in Fig. S4), even though there is no change in upstream IGF-1R activity.

This suggests that structural perturbations in LRs persist even after psychosine is removed, resulting in altered patterns of Akt activation. In summary, the deregulation in Akt phosphorylation is a result of a rapid redistribution of its upstream kinases on the plasma membrane due to disruption of the LR structure by psychosine (White et al., 2009). Hence, psychosine accumulation leads to an uncoupling of receptor phosphorylation from downstream Akt pathway activation by interfering with the proper recruitment of key intermediate kinases to LRs.

### The loss of IGF1-R pathway components from brain lipid rafts in pre-symptomatic twitcher mice

**Fig. 5.**
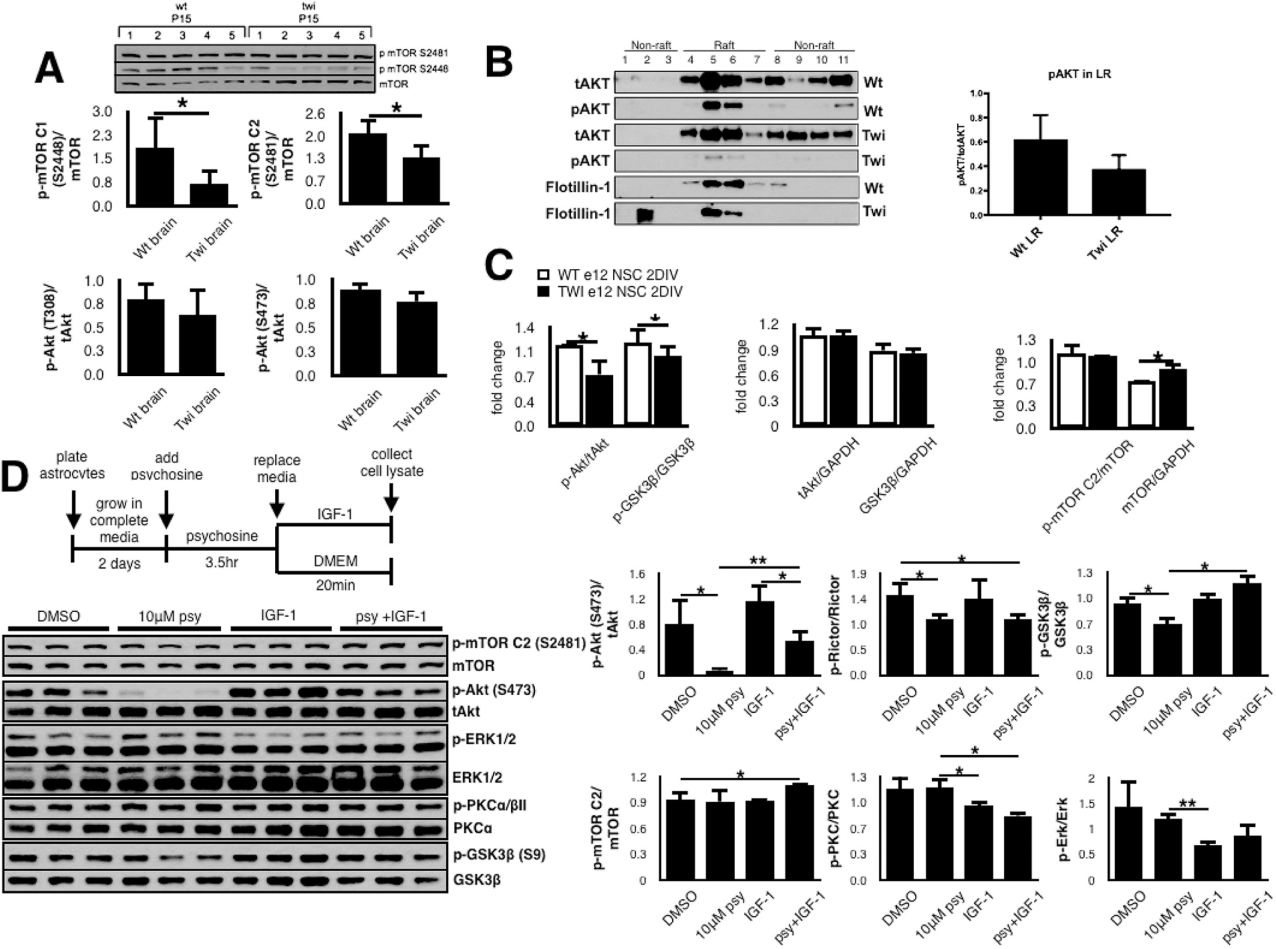
***In vivo* effects of long-term psychosine accumulation on the IGF-1R pathway.** (A) Brains were collected from wild-type (Wt) and twitcher (Twi) mice at postnatal day 15 (P15) and homogenized to obtain whole-cell lysates. An equal amount of proteins was run from each lysate on a western blot and analyzed for p-Akt and p-mTOR. Resulting bands were quantified using the NIH ImageJ software, and the ratios of phosphorylated versus total protein were plotted as mean±s.d., *N*=5 per genotype, *P<0.05, Student's *t*-test. mTORC1 (S2448) and mTORC2 (S2481) levels are decreased in pre-symptomatic twitcher brains. (B) Lipid rafts (LRs) were isolated from whole brains at postnatal day 20 (P20), and the levels of Akt phosphorylation in wild-type and twitcher were determined by immunoblotting. LR structure is disrupted in brains of pre-symptomatic twitcher mice (see flotillin-1), and the level of p-Akt is decreased within twitcher rafts (not statistically significant; data represent mean±s.d., *N*=3, Student's *t*-test). (C) Inhibitory effects of psychosine are visible in twitcher cells as early as the embryonic stage. Neural stem cells were isolated from telencephalons at embryonic day 12 (E12) from wild-type and twitcher embryos, grown as neurospheres and differentiated into mixed glial cultures for 2 days *in vitro* (2 DIV). At the end of differentiation, whole-cell lysates were prepared and subjected to immunoblotting. Bars represent mean±s.d., *N*=3, Student's *t*-test, *P<0.05. Twitcher cultures show a decrease in Akt phosphorylation concurrent with abnormal activation of its target (GSK3β). (D) Primary glia isolated from wild-type pups (P3) were enriched for astrocytes and treated with 10 μM psychosine for 3.5 h followed by 20 min IGF-1 (100 ng/ml) stimulation in the absence of psychosine. Psychosine causes a significant and specific downregulation of the Akt pathway in astrocytes, which can be rescued by IGF-1 treatment. Quantification of immunoblots was performed using NIH ImageJ software. The data represent mean±s.d., *N*=3,  Student's *t*-test, *P<0.05, **P<0.01.

Preferential accumulation of psychosine in LRs of twitcher mice, a naturally occurring KD model, correlates with a decrease in PKC phosphorylation within these domains (White et al., 2009). Given our data from exogenously treated cells, we next asked whether there would be a similar effect on Akt signaling from endogenous psychosine accumulation *in vivo*. Since effects in NCS34 cells are visible soon after psychosine exposure, we hypothesized that a dysregulation in signaling would start at a much earlier time point at the cellular level, before symptoms presented themselves visibly at the peak of myelination after postnatal day 20 (P20). For this, levels of Akt and mTOR phosphorylation were determined in protein extracts from brains of wild-type and pre-symptomatic twitcher mice at P15 (Fig. 5A). There was a small but significant reduction in overall mTORC1 and mTORC2 phosphorylation in whole-brain lysates; however, this reduction did not translate to a decrease in overall p-Akt levels. As we had determined earlier, analysis of total cell lysates could miss specific effects at the membrane; therefore, we compared LRs isolated from brains of twitcher and wild-type mice. Brains of wild-type and pre-symptomatic twitcher mice were subjected to the same detergent-free LR isolation used for NSC34 cells, and components of the IGF-1R pathway were analyzed using western blotting. Similar to NSC34 cells, the LR marker flotillin-1 was redistributed to low-density fractions in twitcher brains, confirming structural disruption (Fig. 5B). In contrast to NSC34 cells, however, IGF-1Rβ and IRS-1 were decreased in twitcher rafts, as well as Rictor, PDK1 and PKCα (data not shown). Although total Akt protein levels within raft and non-raft regions were comparable between wild-type mice and twitchers, there was a reduction in Akt phosphorylation within twitcher rafts (statistically not significant). This effect was not specific to Akt, however, as the levels and phosphorylation of ERK1/2 were also decreased (data not shown), suggesting that both of these survival pathways downstream of IGF-1R are affected.

## DISCUSSION

**Fig. 6.**
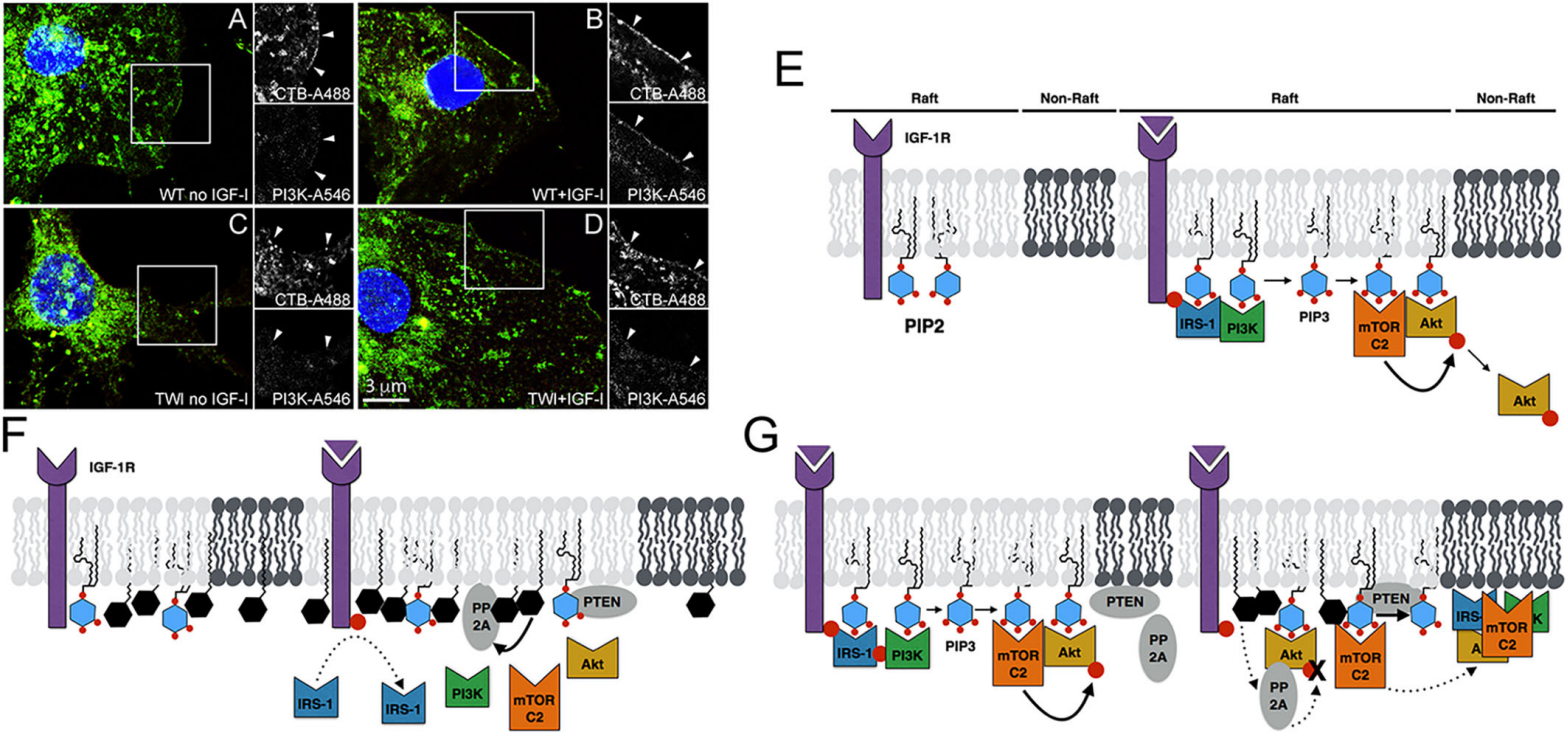
**A model for psychosine toxicity in neurons.** (A-D) Neuronal progenitors from wild-type (WT) and twitcher (TWI) mice were grown under differentiation conditions for 7 days *in vitro* (7 DIV), and cultured in the presence or absence of IGF-1 (100 ng/ml) for 24 h. IGF-1 stimulation of wild-type cells recruits PI3K to lipid rafts (LRs), which are marked by cholera-toxin (arrowheads) (A,B); this effect is absent in twitcher cells (C,D). (E) A working model for psychosine toxicity: activation of the IGF-1R pathway in motor neurons. In unstimulated cells (left panel), IGF-1R and PIP2 reside in LR domains; however, other pathway components are not associated with these microdomains. Upon IGF-1 ligand (purple triangle) binding (right panel), IRS-1 is recruited to the plasma membrane, where it binds phospho-receptors (red circles) via its phosphotyrosine-binding (PTB) domain, and PIP2 on the lipid bilayer via its PH domain. IRS-1 binding leads to the recruitment of PI3K to the plasma membrane, where it converts PIP2 to PIP3. PIP3 in turn recruits mTORC2 and Akt to LRs, putting them in close proximity and causing conformational changes that allow Akt phosphorylation to take place. Once phosphorylated, Akt dissociates from the membrane to activate its cellular targets. (F) Exclusion model. Psychosine (black hexagons) pre-treatment of unstimulated cells leads to its accumulation in PIP2-rich LRs, disrupting its structure (left panel). In addition to rigidifying the membrane, psychosine's positive charge may also act to neutralize the membrane's overall negative charge, or sequester PIP2 molecules through electrostatic interactions influencing local levels. Upon ligand binding to the receptor (right panel), psychosine-mediated disruption in the local microenvironment prevents IRS-1 and PI3K recruitment to the receptor despite its normal activation. In the absence of PI3K, a reduction in local PIP3 generation prevents the recruitment of mTORC2 and stimulation of its activity within rafts, leading to defective Akt phosphorylation. (G) Dispersion model. IGF-1 stimulation of cells leads to normal pathway activation (left panel). Further treating these cells with psychosine in the absence of continued IGF-1 (right panel) puts a brake on pathway activity by dispersing active kinases out of rafts, leading to the blurring of raft and non-raft boundaries. The void left by the moving out of positive regulators may allow for negative pathway regulators to move in their place, such as PTEN and PP1/PP2A, further shutting down the pathway. If psychosine exposure continues for a longer period of time, this scenario would revert back to the conditions in panel F, i.e. even if cells are stimulated with IGF-1 a second time, prior psychosine accumulation prevents a new round of PI3K/mTORC2 from coming in and activating Akt.

In this study, we provide direct evidence that accumulation of the GSL psychosine in the plasma membrane of neuronal cells results in the failure to recruit key kinases to LRs, and leads to the inhibition of multiple downstream pathways essential for neuronal survival. By studying IGF-1R activation, our study demonstrates that psychosine uncouples downstream Akt phosphorylation by interfering with the recruitment of the upstream kinases PI3K and mTORC2 to LRs. A defect in Akt phosphorylation at the membrane has downstream effects on the regulation of its cellular targets, including GSK3β, mTORC1, p70S6K1, and potentially its nuclear targets (Figs 3 and 6).

Our results show that a key step in psychosine neurotoxicity is the inhibition of PI3K recruitment to LRs after IGF-1R is activated (Fig. 6A-D). This presumably originates from a lack of IRS-1 recruitment to raft-resident activated receptors. Since IRS-1 transmits IGF-1 signals to both PI3K-Akt and Ras-ERK, this could explain the decrease of both pathways in our culture studies. Although Ras-MAPK was not part of this study, we speculate that three scenarios could contribute to PI3K recruitment defects leading to Akt inhibition. First, psychosine-mediated changes in membrane properties such as rigidity and/or curvature could modulate the physical binding of these proteins to activated receptors (Carquin et al., 2016; D'Auria and Bongarzone, 2016; D'Auria et al., 2017). Second, psychosine's positive charge could affect the local receptor microenvironment perhaps by sequestering anionic phosphatidylinositol (4,5)-bisphosphate [PtdIns(4,5)*P*_2_; PIP2] through electrostatic interactions, limiting its movement and/or conformational rotation to accommodate PH-domain proteins (McLaughlin et al., 2002). PIP2-rich microenvironments are necessary to recruit a number of PH-domain proteins, including IRS-1 and Akt (Lim et al., 2010). Third, an excess of positively charged psychosine could be countering the membrane's overall negative charge in addition to its membrane rigidification. Psychosine's positive charge is required for its toxicity (Folts et al., 2016) and the anionic nature of the plasma membrane is essential to carry out its function (Srivastava and Voth, 2014), as there is no recruitment of PH-domain proteins if the negative charge is eliminated.

A lack of mTORC2 activation in LRs is the immediate step that leads to Akt inhibition. PI3K is necessary for receptor tyrosine kinase (RTK)-mediated mTORC2 activation, and the binding of PIP3 directly to one of the mTORC2 subunits relieves its auto-inhibition, stimulating its kinase activity towards Akt (Saxton and Sabatini, 2017; Liu et al., 2015; Gan et al., 2011)*.* In our study, we also provide evidence that mTORC2 recruitment and activation at the plasma membrane in response to IGF-1 stimulation is spatially regulated by increased PIP3 levels within rafts. We propose a mechanism where psychosine inhibits mTORC2 and Akt phosphorylation by interfering with local PIP3 generation at the membrane by excluding PI3K from LRs. We offer two alternative, but overlapping, mechanisms (exclusion versus dispersion) on how psychosine could inhibit mTORC2 activity based on our observations from the two conditions that we have tested: in cells treated with psychosine prior to IGF-1 stimulation versus treated with psychosine after IGF-1 stimulation. Although these conditions correspond to the inhibition of initial activation (Fig. 6F) and downregulation of existing pathway activity (Fig. 6G), respectively, one common feature might be the blurring of raft/non-raft boundaries in cells exposed to psychosine.

In the exclusion model (Fig. 6F), psychosine pre-treatment of unstimulated cells leads to its accumulation in LRs. The resulting biophysical changes in membrane architecture prevent the initial recruitment of IRS-1 and PI3K to rafts downstream of IGF-1R activation. In the absence of PI3K, a reduction in local PIP3 generation prevents the recruitment of mTORC2 and stimulation of its activity in LRs, leading to defective Akt phosphorylation. This model is supported by our observations that: (1) IGF-1R phosphorylation is intact in psychosine-treated cells yet PI3K recruitment to LRs is impaired, implying that membrane PIP3 generation is decreased (Figs 4 and 6A-D); (2) increasing PIP3 membrane levels by inhibiting lipid phosphatases that convert it to PIP2 rescues Akt phosphorylation (Fig. S1G); (3) mimicking increased cytoplasmic PIP3 levels using SC-79 is sufficient to bypass inhibition at the plasma membrane and stimulate mTORC2 activity towards Akt (Fig. 2B); (4) inhibition of mTORC2 activation by psychosine specifically takes place at the cell membrane (Fig. 3); and (5) suboptimal doses of psychosine and the PI3K/p110α catalytic inhibitor wortmannin have additive effects on Akt inhibition when combined (Fig. 2A). The final point suggests that psychosine modulates a separate but parallel aspect of PIP3 availability to downstream effectors, either by further decreasing local PIP3 levels by excluding remaining p110α kinases from LRs, and/or limiting access to the PIP3 resident pool by excluding mTORC2 from LRs. These observations suggest that a modulation in PIP2:PIP3 ratio within LRs may be a key aspect of mTORC2/Akt inhibition by psychosine. Psychosine was previously shown to inhibit PLC and interfere with phosphoinositide hydrolysis into IP3 and DAG in primary astrocytes (Ritchie et al., 1992). These observations, combined with the slight increase that we see in PTEN localization to LRs in cells treated with psychosine (Fig. 4B), are further evidence that psychosine most likely acts at the level of PIP2/PIP3. It is notable that DAG activates PKCα and therefore a shift in phosphatidylinositol balance could be an additional cause for PKC inhibition by psychosine besides its defective LR recruitment.

In the dispersion model (Fig. 6G), in which cells are exposed to psychosine after stimulation with IGF-1, we propose that psychosine leads to a shutdown of pathway activity by dispersing active kinases out of rafts and preventing new ones from coming in. Since mTORC2 would no longer be residing in LRs, phosphorylation of new Akt molecules would not take place. The void left by the outward movement of positive regulators may allow for negative regulators to move into their place in LRs, such as PTEN and PP1/PP2A, further shutting down the pathway. Other sphingolipids are known to cause the movement of receptors out of rafts with corresponding changes in downstream signaling (Pituch et al., 2015); however, receptor movement does not seem to be involved in our system. Although we do not know why proteins disperse out of rafts, it could be because of physical disruption, a lack of PIP3 generation, or a smearing of raft/non-raft boundaries due to excessive psychosine insertion. This smearing effect might also explain why we detect ectopic Akt phosphorylation in non-rafts when psychosine is removed from media prior to IGF-1 stimulation (Fig. S4).

Finally, mTORC1 and mTORC2 have diverse functions within the cell that are tightly regulated by nutrients and GFs (Saxton and Sabatini, 2017). Unlike mTORC1, whose regulation has been extensively studied, upstream regulation of mTORC2 is largely unknown. We show that GF-dependent recruitment of mTORC2 to neuronal LRs is necessary for its kinase activity towards Akt. To our knowledge, this is the first time mTORC2 has been shown to be phosphorylated at S2481 in an LR-dependent manner in any cell type. We also show for the first time that Rictor, an essential mTORC2-specific subunit, is phosphorylated within both LRs and the nucleus in a GF-dependent manner. This study, along with earlier observations regarding sphingolipid effects on established mTOR functions, therefore provides a previously unknown connection between the regulation of mTORC2 activity by PIP3 within LRs and a genetic nervous-system disease. An outstanding question from our work is to determine whether this LR connection would also have implications in the context of other neurodegenerative conditions and cancer.

In conclusion, our work predicts that pathway activities that rely mainly on LR integrity in any given cellular context would be affected by psychosine treatment, especially GF-mediated RTKs and chemokine-mediated G protein-coupled receptors. Examples include IGF-1-mediated Akt activation in muscle cells, TGFβ-mediated ERK activation in epithelial cells, or LR-mediated signaling in neuroinflammation. We hypothesize that, depending on which pathways are active in any given cell type at any given time, the ones that depend significantly on LRs for activation and/or continued activity will be sensitive to psychosine exposure.

### Perspectives for improving KD therapies

The fact that psychosine exposure does not completely shut down Akt phosphorylation, and that IGF-1R pathway components are intact and inhibition is reversible under certain conditions, offers a window of opportunity for therapeutic interventions. An attractive idea for therapy would be to treat patients with IGF-1 and/or other GFs to improve intracellular signaling and therefore cellular resilience to psychosine. We believe that this treatment should be done in conjunction with hematopoietic stem cell transplantation or gene therapy, and continued for a specified period of time after genetic correction. Persistence of psychosine in rafts is a limiting factor for therapeutic effectiveness in mouse models of KD (White et al., 2011), suggesting that GALC activity should be restored as much as possible to minimize psychosine-driven pathogenesis in neurons before IGF-1 treatment can work.

Additionally, we believe that treatment should begin very early to be effective. We see negative effects on cellular signaling soon after the cells are exposed to psychosine (Fig. 1A), and these effects are not limited only to neurons since psychosine treatment of wild-type astrocytes also shows a similar downregulation of Akt phosphorylation that can be rescued with IGF-1 treatment (Fig. 5D). In addition, our preliminary data in differentiated neural stem cells from twitcher embryos suggest that the PI3K-Akt-mTOR pathway is defective as early as embryonic day (E)12 (Fig. 5C, Table S2), and imply that neurogenesis, and possibly other developmental processes, could be affected during this period *in utero*. There have been cases of some infants already born with symptoms, and by this point the damage is irreversible and these infants are no longer candidates for transplantation. Therefore, it may be essential for combination therapies to be initiated at the earliest time point possible.

In conclusion, surmounting evidence suggests that psychosine exerts its effects mainly through structural disruption of LRs rather than specific psychosine-protein interactions, with its demonstrated interaction with α-synuclein being an exception (Smith et al., 2014; Marshall and Bongarzone, 2016; Abdelkarim et al., 2018). We hypothesize that psychosine initiates a domino effect at the membrane that affects many downstream cellular processes. Future therapies may benefit from drug screens directed at more upstream targets at the membrane level, which may include membrane-stabilizing strategies to protect membrane structure and integrity.

## MATERIALS AND METHODS

### Ethics statement

The use of twitcher mice and the experimental studies were approved by the Animal Care and Use Committee of the University of Illinois at Chicago (protocol number 15–101). Breeder twitcher heterozygous mice (C57BL/6J, twi/+) were originally purchased from Jackson Laboratory, maintained and treated under standard housing conditions using the Animal Care and Use Committee protocols of our institution. Male and female twitcher mice were used indistinctly. Mice were anesthetized by isofluorane, and killed by cervical dislocation.

### Neural progenitor cultures

Neural progenitors were isolated from wild-type and twitcher embryonic telencephalons at E12 according to previously published protocols (Pituch et al., 2015). Neural progenitors were grown as proliferative neurospheres in Neurocult media (STEMCELL, cat. no. 05700) in the presence of proliferation supplement (cat. no. 05702) and the GFs EGF (20 ng/ml) and bFGF (10 ng/ml) according to the manufacturer’s protocol. Neurospheres (passage 8) were dissociated, plated on Matrigel-coated 6-well plates (0.5×10^6^ cells/well), and differentiated into mixed glial cultures for 2 days *in vitro* (DIV) using Neurocult media containing differentiation supplement (cat. no. 05704). Whole-cell lysates were prepared in RIPA buffer with protease and phosphatase inhibitors. An equal amount of protein was analyzed from wild-type and twitcher cultures on a western blot.

### Immunocytochemistry

Neuronal progenitors from wild-type and twitcher mice were grown under differentiation conditions for 7 DIV, and cultured in the presence or absence of IGF-1 (100 ng/ml) for 24 h. Cells were fixed in 4% paraformaldehyde/PBS and then blocked in 5% BSA/PBS. Cells were incubated with 1:100 dilution of cholera-toxin–Alexa-Fluor-488 (Molecular Probes) and 1:100 dilution of rabbit anti-PI3K (Cell Signaling) at 4°C overnight. After incubation with anti-rabbit IgG-Alexa-Fluor-546 (Molecular Probes; 1:1500 dilution in blocking buffer), samples were confocally imaged in an upright Leica TCS DM5500Q confocal microscope (Leica Biosystems, Buffalo Grove, IL, USA).

### NSC34 cell culture and treatments

Details of the NSC34 cells used in this study were previously published elsewhere (Castelvetri et al., 2011). NSC34 cells were grown as a monolayer in a humidified incubator at 37°C in 5% FBS/DMEM and passaged as a single-cell suspension every 3 days until 70% confluent. To minimize variation during experimental treatments at different times, a large batch of cells from the same parent culture at passage 27 was frozen in multiple vials using DMSO. We found that earlier-passage cells were more resistant to the effects of psychosine; therefore, later-passage cultures were used. For each subsequent experiment, one vial was thawed from this batch at passage 27, passaged once at passage 28 for expansion, and plated at  passage 29 in 6-well plates (for psychosine and inhibitor experiments) or T75 flasks (for subcellular fractionation) before treatment. For example, the individual frozen vial of cells used during an experiment for treatment with psychosine in a 6-well plate is from the same batch as the vial used to set up the subcellular fractionation experiments, or the LR isolations. For psychosine and inhibitor treatments, 5×10^4^ NSC34 cells were plated on Matrigel-coated 6-well plates (day 0) and grown in 5% FBS/DMEM for 3 days until 60% confluent (day 3). If conditions did not require serum deprivation, cells were washed with warm 1× PBS on day 3 and treated with psychosine working solution with the indicated concentrations (in DMEM; see figures) at 37°C for the indicated times (0.1% DMSO final). If conditions required serum deprivation, cells were washed with warm 1× PBS on day 3, and serum-free DMEM was added for 24 h. Each treatment condition was performed in triplicate on day 4 (i.e. the same condition was used in three separate wells on a 6-well plate). After treatment, cells were washed in cold 1× PBS and 80 μl of ice-cold RIPA lysis buffer (Sigma-Aldrich, cat. no. R0278) containing protease/phosphatase inhibitors (Pierce™ Protease Inhibitor Mini Tablets, EDTA-free, cat. no. 88666, Pierce™ Phosphatase Inhibitor Mini Tablets, cat. no. 88667) were added in each well and incubated on ice for 15 min. Lysates were transferred to an Eppendorf tube and centrifuged at 16,000 ***g*** for 30 min, and supernatant was transferred to a fresh tube. Protein concentrations were determined using the Pierce MicroBCA Protein Assay kit (cat. no. 23235). To analyze phosphorylation levels, an equal amount of protein (5-7.5 μg) from each triplicate per condition was pooled and run in a single lane on a 4-12% NuPAGE Bis-Tris protein gel (Invitrogen). This was repeated at least three times for each condition, representing the average of nine data points per condition. See Table S1 for a complete list of inhibitors and reagents, and the concentrations that were used.

### NSC34 subcellular fractionation

Subcellular fractionation of NSC34 cells was performed using the Thermo Fisher Scientific Subcellular Protein Fractionation Kit for Cultured Cells (cat. no. 78840) according to the manufacturer's protocol. A total of 750,000 NSC34 cells were transferred to a T75 flask and grown in complete media (5% FBS/DMEM) for 3 days until 50-60% confluent, and serum deprived in DMEM for 24 h. Following treatment, cells were dissociated from the flask using Cellstripper solution (CellGro, cat. no. 25-056-CI), 5 ml cold DMEM (no FBS) was added and cell suspension was transferred to a 15 ml Falcon tube. Cells were centrifuged at 4°C, 1000 rpm for 5 min, and the supernatant was discarded. The pellet was resuspended in 1 ml cold PBS; 100 μl was transferred to an Eppendorf tube and saved for INPUT (to obtain whole-cell lysate); the remaining cell suspension (900 μl) was transferred to another Eppendorf and centrifuged at 4°C, 500 ***g*** for 2-3 min. For whole-cell lysates, cell pellet from the INPUT fraction was processed with RIPA buffer containing protein and phosphatase inhibitors, and vortexed periodically to lyse the cells while being kept on ice. Cells were then centrifuged at 16,000 ***g*** for 10 min and supernatant was saved as INPUT whole-cell extract (WCE). To obtain the cytoplasmic fraction, cold cytosol extraction buffer (CEB) with protease and phosphatase inhibitors was added to the remaining pellet (∼20 μl packed volume) and incubated for 10 min at 4°C while rotating. Cells were centrifuged at 500 ***g*** for 5 min at 4°C and supernatant was collected as the cytoplasmic extract (CE). The CE was centrifuged once again at 16,000 ***g*** for 10 min at 4°C to further clear the fraction. Cold membrane extraction buffer (MEB) (+inhibitors) was added to the pellet, vortexed for 5 s and incubated at 4°C for 15 min while rotating. Lysate was centrifuged at 3000 ***g*** for 5 min at 4°C and supernatant was collected as the membrane extract (ME). Cold nuclear extraction buffer (NEB) (+inhibitors) was added to the pellet, vortexed for 15 s and incubated for 30 min at 4°C while rotating. Lysate was centrifuged at 5000 ***g*** for 5 min at 4°C and supernatant was collected as the soluble nuclear extract (SNE). Protein concentration in each fraction was determined with the Pierce MicroBCA Protein Assay kit (cat. no. 23235) and 10 μg protein from each fraction for each condition was run on a western blot for comparison.

### Western blot protocol

Samples were run on a 4-12% NuPAGE Bis-Tris protein gel (Invitrogen) using Invitrogen NuPAGE™ MOPS SDS Running Buffer (cat. no. NP000102) for 2.5 h at 120 V, and proteins were transferred onto PVDF membrane in a wet transfer tank using Invitrogen NuPAGE™ Transfer Buffer (cat. no. NP00061) (without methanol) for 2 h at 350 mA. After the wet transfer, PVDF membranes were transferred to a container and covered in 100% methanol, and left to dry overnight at room temperature. In our experience, this helped with the binding of proteins to the membrane surface and gave better chemiluminescent signals. PVDF membranes were then cut along molecular mass markers into multiple pieces containing distinct proteins of interest (e.g. cut between the 50 kD and 75 kD marker lines to obtain a section containing the Akt protein; above the 200 kD marker line to obtain a section containing the mTOR protein, etc., from the same PVDF membrane). When ready for blotting, PVDF sections were re-activated briefly in methanol, and blocked in 5% milk at 4°C overnight. For comparing phosphorylated versus total protein (e.g. p-Akt versus total Akt), membranes were first incubated with the phospho-antibody, stripped in stripping buffer and re-probed with an antibody for the non-phospho protein. Band intensities were quantified using the freely available NIH ImageJ software. For a complete list of antibodies and reagents used for the study, please see Table S1. For antibody validation profiles, please refer to the manufacturer’s website.

### Isolation of lipid-raft/caveolae

#### NSC34 cells

A total of 1×10^6^ NSC34 cells were seeded in a T175 flask and grown in complete media for 3 days until 50-60% confluent, washed twice with warm 1× PBS and serum deprived for 24 h. Following treatment, cells were immediately placed on ice and scraped in detergent-free Tricine buffer (250 mM sucrose, 1 mM EDTA, 20 mM Tricine, pH 7.4). Cellular material was homogenized and centrifuged at low speed (1400 ***g*** for 5 min at 4°C) to precipitate nuclear material. Resulting supernatant [homogenate (H)] was collected, mixed with 30% Percoll in Tricine buffer and subjected to ultracentrifugation for 25 min (Beckmann MLS50 rotor, 77,000 ***g*** at 4°C) to collect the plasma membrane fraction (PM). PMs were collected and sonicated (3×3-s bursts). The sonicated material was mixed with 60% sucrose (to a final concentration of 40%), overlaid with a 5-35% step sucrose gradient and subjected to overnight ultracentrifugation (Beckman MLS50 rotor, 87,400 ***g*** at 4°C). Fractions were collected every 400 μl from the top sucrose layer and proteins were precipitated using 0.25 volume TCA–deoxycholic-acid in double-distilled water [0.1% (wt.vol) deoxycholic acid] to precipitate proteins. Protein pellets were washed twice with 100% acetone and air dried. Pellets were resuspended in an equal amount of loading buffer; an equal amount of each fraction was run in separate lanes on a western blot.

#### Twitcher tissue

Brains were collected from P20 wild-type and homozygous twitcher mice, homogenized in Tricine buffer, and processed as above. Male and female twitcher mice were used indistinctly.

### Mouse brain tissue collection and preparation of total lysates

Brain tissue was collected, immediately immersed in liquid nitrogen and stored at −80°C until further use. Male and female twitcher mice were used indistinctly. Tissues were thawed on ice, quickly rinsed in ice-cold 1× PBS and processed to obtain whole-cell lysates by homogenization in RIPA buffer (Sigma-Aldrich, cat. no. R0278) containing protein and phosphatase inhibitors (Pierce™ Protease Inhibitor Mini Tablets, EDTA-free, cat. no. 88666; Pierce™ Phosphatase Inhibitor Mini Tablets, cat. no. 88667). Lysates were incubated on ice for 30 min and vortexed every 10 min. Lysates were centrifuged at 16,000 ***g*** for 15 min and the supernatant was transferred to a fresh tube and centrifuged again to clear the lysate at 16,000 ***g*** for 30 min. Supernatant was transferred to another tube and protein concentrations were determined using the Pierce MicroBCA Protein Assay kit (cat. no. 23235).

### Microwestern array

Neural progenitors were isolated from wild-type and twitcher spinal cords at E12 and grown as neurospheres according to previously published protocols (Pituch et al., 2015). Neurospheres (passage 4) were dissociated, plated on Matrigel-coated Petri dishes and differentiated into mixed-glial cultures for 7 DIV until near confluence (Table S2A)*.* Differentiated cells were sent to the Microwestern Array Core Facility (MWAC) at The University of Chicago, USA, where cell extracts were blotted for quantitative analysis of an array of pre-selected antibodies (Table S3). Each antibody was tested in duplicate per sample. For further details on microwestern array methodology, please refer to (http://www.igsb.org/services/mwac-methodology).

### Psychosine treatment of primary astrocytes

Primary glia were isolated from the cortex of wild-type myelin proteolipid protein (PLP)-GFP pups at postnatal day 3, and grown in glia media according to published protocols (Cole and de Vellis, 2001). Glial cultures were shaken to remove microglia/oligodendrocytes, and remaining cells enriched for primary astrocytes were transferred to T75 flasks. On day 8, flasks were shaken to remove oligodendrocytes, and remaining astrocytes were plated in Matrigel-coated 6-well plates on day 12. Psychosine treatment was done on day 14 in enriched astrocyte cultures and cell lysates were collected in RIPA buffer. A total of 10 μg protein from each sample was run on a western blot.

### Statistical analysis

No pre-specified effect size was assumed in experiments. For mouse experiments, 3-5 mice per genotype were used. For cell culture, experiments were repeated at least three times with three technical replicates each. Values are mean±s.d. of at least three independent experiments. Statistical analysis was done using Student's *t*-test. *P<0.05; **P<0.01; ***P<0.001.

## Supplementary Material

Supplementary information
