# Supplementary material for: Inhibition of the IGF-1–PI3K–Akt–mTORC2 pathway in lipid rafts increases neuronal vulnerability in a genetic lysosomal glycosphingolipidosis
Source: Dis Model Mech. 2019 May 23;12(5):dmm036590. doi: 10.1242/dmm.036590 (PMC6550048; doi:10.1242/dmm.036590)
Supplement: Supplementary information [file dmm-12-036590-s1.pdf]

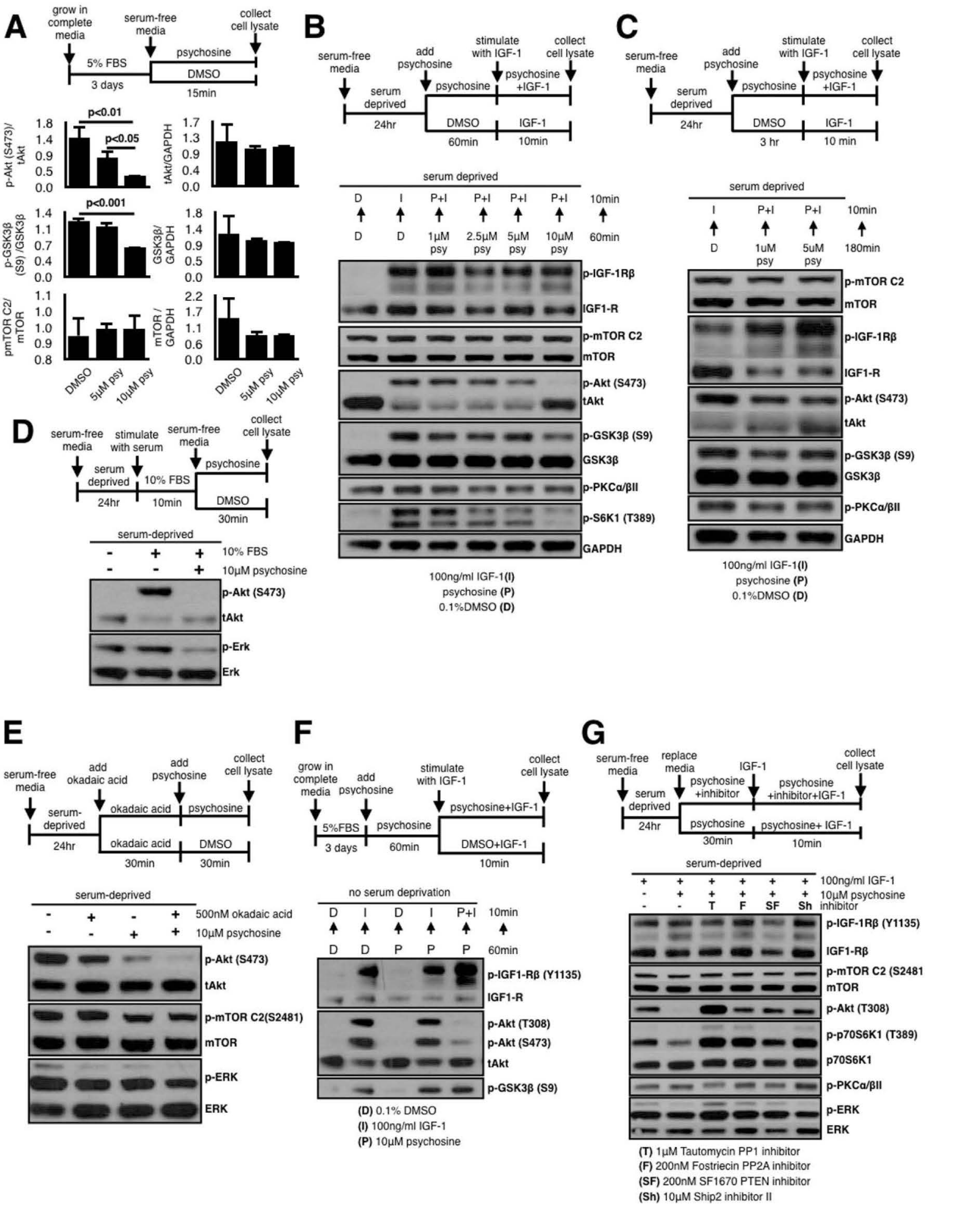

Disease Models & Mechanisms • Supplementary information

Figure S1. *Psychosine interferes with growth factor stimulation of p-Akt in a time and dose dependent manner.* **(A)** Cells were grown in complete media until 60% confluent and treated with 0.1% DMSO, 5µM or 10µM psychosine in serum-free media for 15 min. Protein levels in whole cell lysates were determined by immunoblotting and quantified using NIH Image J. The data represents mean ± SD from technical triplicate samples from one experiment and is representative of additional experiments not shown. Akt pathway inhibition by psychosine is rapid and dose-dependent. **(B)** Cells were serum deprived for 24 hr and pre-treated with increasing doses of psychosine in serum-free media for 1 hr. Treated cells were subsequently stimulated for 10 min with IGF-1 (100ng/ml) in the presence or absence of continued psychosine at the same concentration. Psychosine inhibits Akt activation in a dose-dependent manner. **(C)** Cells were serum deprived for 24 hr and pre-treated with 1µM or 5µM psychosine in serum-free media for 3 hr. Treated cells were subsequently stimulated with IGF-1 (100ng/ml) in the presence or absence of continued psychosine. Inhibition of Akt phosphorylation by psychosine is cumulative over time.**(D)** Cells were serum deprived for 24 hr and stimulated with 10% fetal bovine serum (FBS) for 10 min, followed by 10µM psychosine in serum-free media for 30 min. Akt phosphorylation, not ERK1/2, responds to serum stimulation in NSC34 cells and is more sensitive to inhibition by psychosine. The image is a representative of at least 3 independent experiments. **(E)** Cells were serum deprived for 24 hr and pre-treated with 500nM okadaic acid (OA) for 30 min to block cellular phosphatase activity under basal conditions. Cells were subsequently treated with 10µM psychosine in serum-free media for 30 min. Unlike IGF-1 stimulated conditions (compare to Fig. 1F), OA fails to rescue psychosine-mediated inhibition of p-Akt under basal conditions. **(F)** Cells were grown in complete media until 60% confluent and treated with 10µM psychosine in serum-free media for 1 hr, followed by 10 min IGF-1 (100ng/ml) stimulation in the presence or absence of psychosine. IGF-1 rescues psychosine-mediated inhibition of p-Akt only when psychosine is absent from media during growth factor stimulation. **(G)** Cells were serum deprived for 24 hr and pre-treated for 1 hr with 10µM psychosine alone, or in combination with indicated specific phosphatase inhibitors. Cells were subsequently stimulated with IGF-1 (100ng/ml) for 10 min under the same pre-treatment conditions. Akt phosphorylation can be rescued by inhibiting any one of the negative regulators of the IGF1-R pathway.

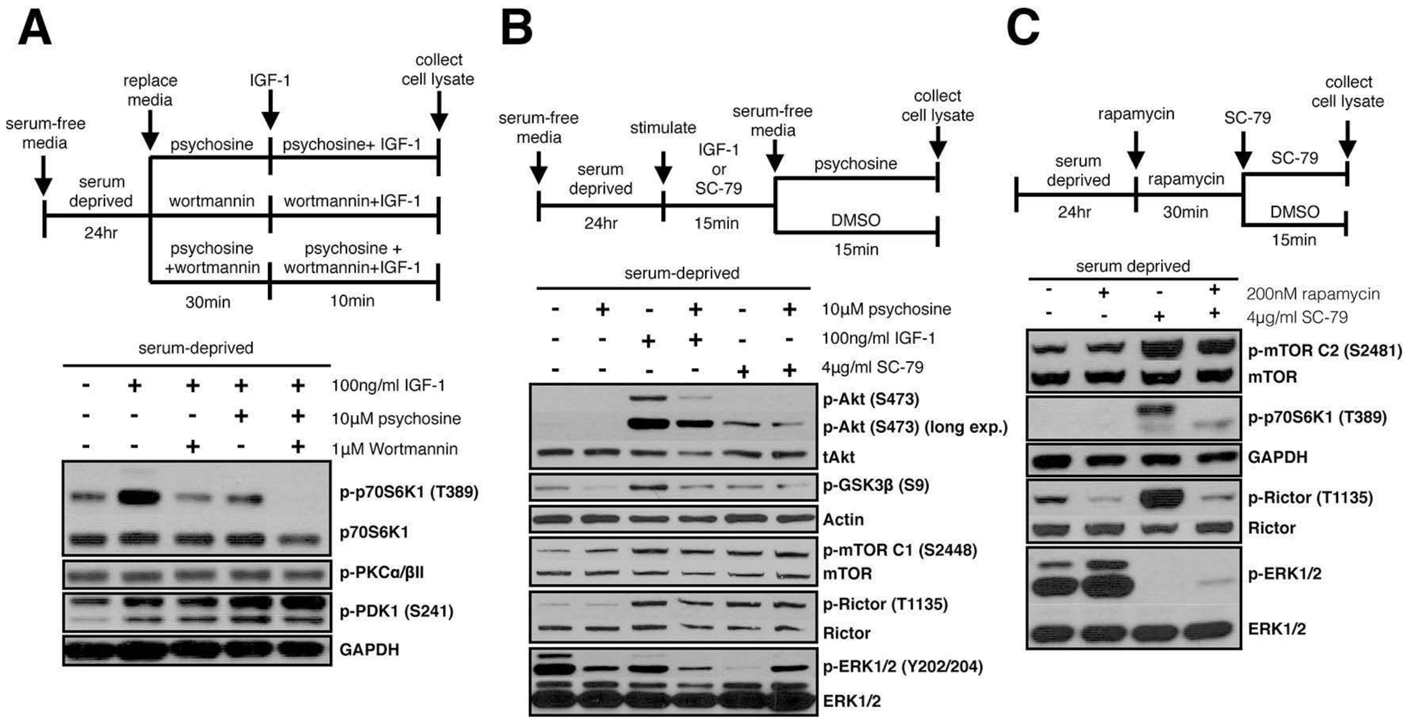

Figure S2. *Membrane-dependent activation of the Akt pathway is more sensitive to psychosine inhibition.* **(A)** Experiment continued from Fig 2A. When combined, psychosine and wortmannin fully inhibit IGF-1 stimulated phosphorylation of p70S6K1 and its immediate target p-Rictor, but not that of mTORC2 target PKCα. **(B)** Side-by-side comparison of p-Akt inhibition when activated at the membrane through IGF-1 stimulation, or within the cytosol by SC-79 treatment. Unlike membrane-stimulated Akt phosphorylation, cytosolic p-Akt is resistant to inhibition by psychosine. **(C)** Cells were pre-treated with rapamycin to inhibit mTORC1/S6K activity, followed by stimulation with the PIP3 analog SC-79. The level of Rictor phosphorylation is a direct readout of mTORC1-mediated p70S6K1 activity, and is independent of mTORC2 auto-phosphorylation at S2481.

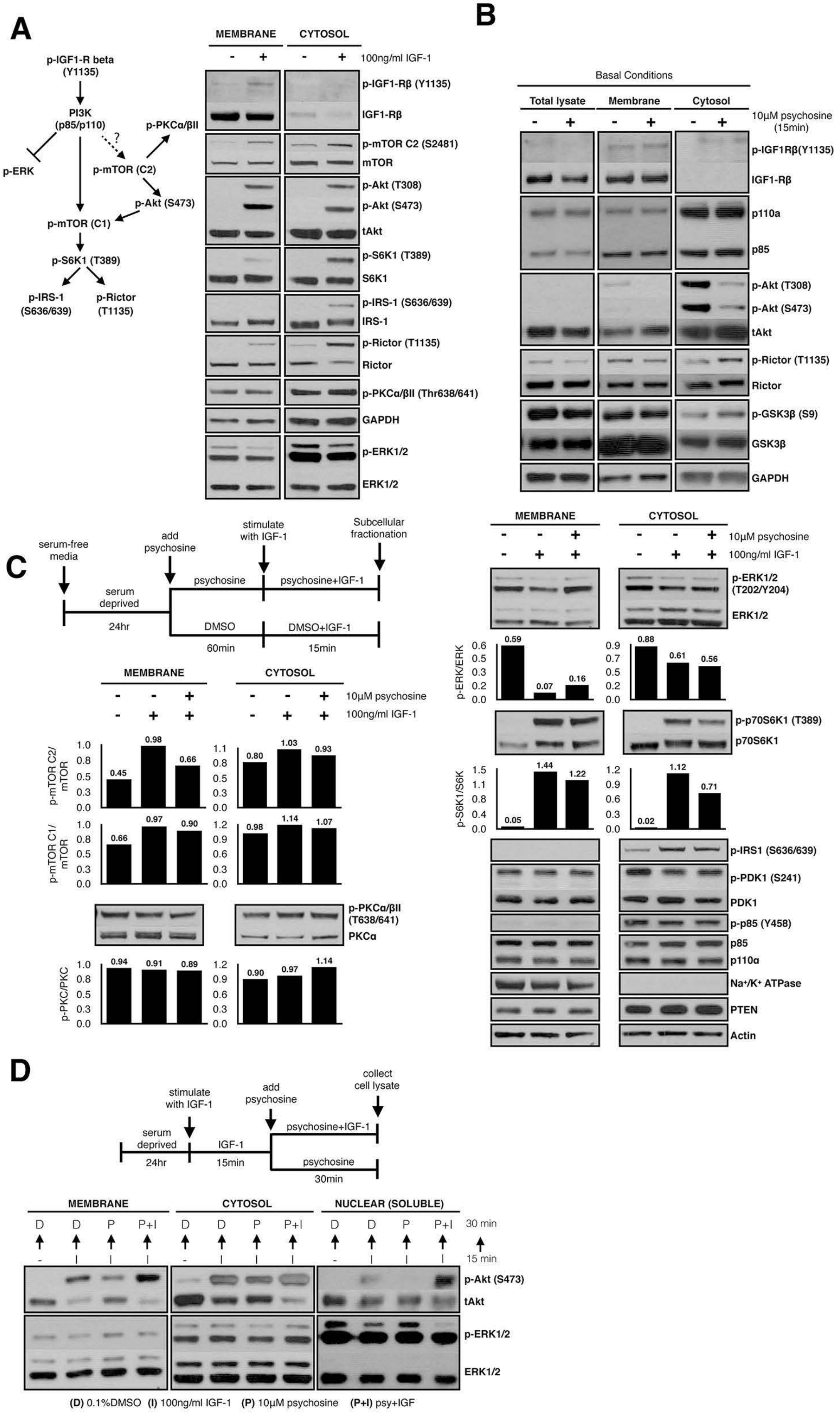
A
p-IGF1-R beta (Y1135)
PI3K (p85/p110)
p-ERK
p-mTOR (C2)
p-PKCα/βII
p-Akt (S473)
p-mTOR (C1)
p-S6K1 (T389)
p-IRS-1 (S636/639)
p-Rictor (T1135)
MEMBRANE
CYTOSOL
100ng/ml IGF-1
p-IGF1-Rβ (Y1135)
IGF1-Rβ
p-mTOR C2 (S2481)
mTOR
p-Akt (T308)
p-Akt (S473)
tAkt
p-S6K1 (T389)
S6K1
p-IRS-1 (S636/639)
IRS-1
p-Rictor (T1135)
Rictor
p-PKCα/βII (Thr638/641)
GAPDH
p-ERK1/2
ERK1/2
B
Basal Conditions
Total lysate
Membrane
Cytosol
10µM psychosine (15min)
p-IGF1Rβ(Y1135)
IGF1-Rβ
p110a
p85
p-Akt (T308)
p-Akt (S473)
tAkt
p-Rictor (T1135)
Rictor
p-GSK3β (S9)
GSK3β
GAPDH
C
serum-free media
add psychosine
stimulate with IGF-1
Subcellular fractionation
serum deprived
24hr
psychosine
psychosine+IGF-1
DMSO
DMSO+IGF-1
60min
15min
MEMBRANE
CYTOSOL
10µM psychosine
100ng/ml IGF-1
p-mTOR C2/ mTOR
0.45
0.98
0.66
0.80
1.03
0.93
p-mTOR C1/ mTOR
0.66
0.97
0.90
0.98
1.14
1.07
p-PKCα/βII (T638/641)
PKCα
p-PKC/PKC
0.94
0.91
0.89
0.90
0.97
1.14
p-ERK1/2 (T202/Y204)
ERK1/2
p-ERK/ERK
0.59
0.07
0.16
0.88
0.61
0.56
p-p70S6K1 (T389)
p70S6K1
p-S6K1/S6K
0.05
1.44
1.22
0.02
1.12
0.71
p-IRS1 (S636/639)
p-PDK1 (S241)
PDK1
p-p85 (Y458)
p85
p110α
Na+/K+ ATPase
PTEN
Actin
D
serum deprived
24hr
stimulate with IGF-1
IGF-1
15min
add psychosine
psychosine+IGF-1
psychosine
30min
collect cell lysate
MEMBRANE
CYTOSOL
NUCLEAR (SOLUBLE)
D
P
P+I
30 min
15 min
p-Akt (S473)
tAkt
p-ERK1/2
ERK1/2
(D) 0.1%DMSO (I) 100ng/ml IGF-1 (P) 10µM psychosine (P+I) psy+IGF

Figure S3. *Psychosine effects on the activation of IGF-1R pathway components in distinct subcellular compartments.* **(A)** Cells were serum deprived for 6 hr to bring phosphorylation down to basal levels, followed by stimulation with 100ng/ml IGF-1 for 15 min. Under basal conditions, Akt protein is mainly cytosolic and has minimal phosphorylation (exposure for each subcellular fraction were adjusted to a similar level to highlight the changes in phosphorylation levels, and does not reflect the relative abundance of the proteins in different fractions). IGF-1 addition leads to receptor phosphorylation and subsequent stimulation of p-mTORC2, p-Rictor and p-Akt at the membrane. **(B)** Cells were serum deprived for 24 hr and treated with 10μM psychosine for 15 min. Psychosine downregulates Akt phosphorylation in the cytoplasm under basal conditions. **(C)** Continued from Fig. 3. Band intensities from immunoblots were quantified using the NIH Image J software. Psychosine affect is more visible at the cell membrane and is specific to only a subset of IGF-1R pathway components. **(D)** Continued from Fig. 1D (total cell lysates). Treated cells were further processed for subcellular fractionation. The inhibitory effect of psychosine on Akt phosphorylation extends from the cell membrane to the nucleus. Continued IGF-1 stimulation overrides this inhibition in all subcellular compartments, and this effect is specific to p-Akt.

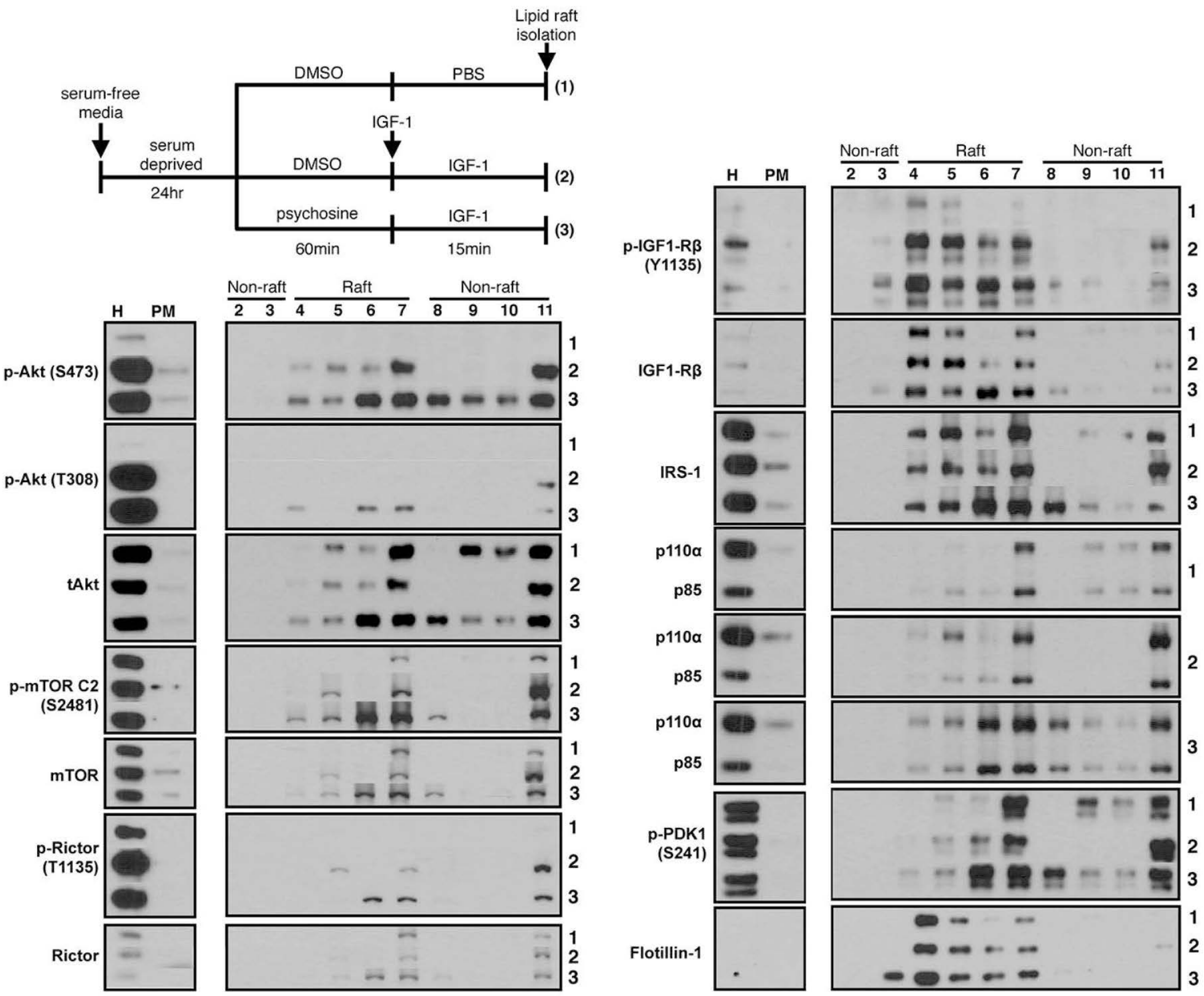

Figure S4. *Psychosine pre-treatment leads to abnormal Akt activation outside of lipid rafts following IGF-1 stimulation.* Cells were serum deprived for 24 hr and incubated with 10μM psychosine for 1 hr. Psychosine was removed from media and cells were stimulated with IGF-1 (100ng/ml) for 15 min in the absence of psychosine. Lipid raft fractions were isolated as in Fig. 4. Treatment with psychosine leads to a redistribution of Akt protein, and increased recruitment of upstream kinases, resulting in abnormal Akt activation in non-raft domains.

Table S1. List of antibodies and reagents used in this study.

Reagent	Company	Catalog No.	Dilution/concentration
DMSO	Sigma-Aldrich	D2650	0.1%
Psychosine (free amine form)	Matreya LLC	SPL1305	1-10µM
Matrigel Matrix Growth Factor Reduced (GFR)	Corning	356230	1:1000
human IGF-1	Peprotech	100-11	100ng/ml
SC-79	Tocris Bioscience	4635	4µg/ml
Tautomycin	Sigma-Aldrich	86305	1µM
Fostriecin	Tocris Bioscience	1840	200nM
PTEN inh. SF1670	Sigma-Aldrich	SML0684	200nM
Rapamycin	Santa Cruz Biotech	SC-3504A	200nM
Wortmannin	Cell Signaling Technologies	9951	0.1µM, 1µM
Ship-2 inhibitor	Calbiochem	508318	10µM
InSolution Okadaic Acid	Calbiochem	495609	250nM, 500nM
Flotillin-1 Santa Cruz	Santa Cruz Biotech	sc-25506	1:1000
Actin	Sigma-Aldrich	A2066	1:3000
GAPDH	Proteintech	60004-1-Ig	1:10000

Disease Models & Mechanisms • Supplementary information

Phospho-PI3 Kinase p85 (Tyr458)/p55 (Tyr199) Antibody	Cell Signaling Technologies	4228	1:1000
PI3 Kinase p110α (C73F8) Rabbit mAb	Cell Signaling Technologies	4249	1:3000
PI3 Kinase p85 (19H8) Rabbit mAb	Cell Signaling Technologies	4257	1:3000
mTOR (7C10) Rabbit mAb	Cell Signaling Technologies	2983	1:3000
Phospho-mTOR (Ser2448) (D9C2) XP® Rabbit mAb	Cell Signaling Technologies	5536	1:3000
Phospho-mTOR (Ser2481) Antibody	Cell Signaling Technologies	2974	1:3000
Rictor (53A2) Rabbit mAb	Cell Signaling Technologies	2114	1:3000
Phospho-Rictor (Thr1135) (D30A3) Rabbit mAb	Cell Signaling Technologies	3806	1:3000
Raptor (24C12) Rabbit mAb	Cell Signaling Technologies	2280	1:3000
Phospho-p70 S6 Kinase (Thr389) (108D2) Rabbit mAb	Cell Signaling Technologies	9234	1:2000
p70 S6 Kinase (49D7) Rabbit mAb	Cell Signaling Technologies	2708	1:2000

Disease Models & Mechanisms • Supplementary information

Phospho-Akt (Ser473) (D9E) XP® Rabbit mAb	Cell Signaling Technologies	4060	1:3000
Phospho-Akt (Thr308) (D25E6) XP® Rabbit mAb	Cell Signaling Technologies	13038	1:3000
Akt (pan) (C67E7) Rabbit mAb	Cell Signaling Technologies	4691	1:3000
Akt (pan) (40D4) Mouse mAb	Cell Signaling Technologies	2920	1:3000
Phospho-GSK-3β (Ser9) (5B3) Rabbit mAb	Cell Signaling Technologies	9323	1:5000
GSK-3β (D5C5Z) XP® Rabbit mAb	Cell Signaling Technologies	12456	1:5000
Phospho-IRS-1 (Ser636/639) Antibody	Cell Signaling Technologies	2388	1:2000
IRS-1 (D23G12) Rabbit mAb	Cell Signaling Technologies	3407	1:3000
PTEN (D4.3) XP® Rabbit mAb	Cell Signaling Technologies	9188	1:1000
Phospho-IGF-I Receptor β (Tyr1135) (DA7A8) Rabbit mAb	Cell Signaling Technologies	3918	1:500

Disease Models & Mechanisms • Supplementary information

IGF-I Receptor β (D23H3) XP® Rabbit mAb	Cell Signaling Technologies	9750	1:1000
Phospho-p44/42 MAPK (Erk1/2) (Thr202/Tyr204) (D13.14.4E) XP® Rabbit mAb	Cell Signaling Technologies	4370	1:3000
ERK1/2 antibody	Cell Signaling Technologies	4695	1:3000
Phospho-PKCα/β II (Thr638/641) Antibody	Cell Signaling Technologies	9375	1:3000
PKCα Antibody	Cell Signaling Technologies	2056	1:2000
Phospho-PDK1 (Ser241) (C49H2) Rabbit mAb	Cell Signaling Technologies	3438	1:3000
PDK1 Antibody	Cell Signaling Technologies	3062	1:2000
Anti-alpha 1 Sodium Potassium ATPase antibody [464.6]	Abcam	ab7671	1:1000
anti-rabbit IgG HRP-linked Antibody	Cell Signaling Technologies	7074	1:3000
anti-mouse IgG HRP-linked Antibody	Cell Signaling Technologies	7076	1:3000

Table S2. *PI3K/Akt/mTOR signaling is downregulated in twitcher cells as early as embryonic day 12.* Neural stem cells were isolated from spinal cords of wild-type and twitcher embryos at embryonic day 12 and grown as neurospheres. Neurospheres (passage 4) were differentiated into mixed glial cultures containing neurons, astrocytes and oligodendrocytes for 7 days in vitro (A). Differentiated cultures were assessed for protein levels (normalized to both tubulin and GAPDH) using the quantitative immunoblotting method Microwestern Array (B) (see materials and methods). First three columns on the left contain proteins and their modifications that were found to be downregulated more than two-fold in differentiated twitcher cultures compared to wild-type. Right two columns contain proteins that were either downregulated less than two-fold, unchanged or upregulated in twitcher cultures compared to wild-type. All of the IGF1-R pathway components that we have analyzed in this study are downregulated by more than two-fold in differentiated twitcher NSCs.

A

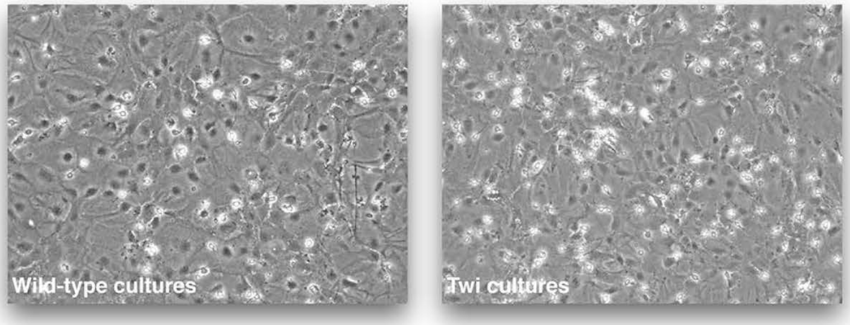

B

Downregulated > 2-fold		
IGFRβ	Caveolin-1	CREB
p85/PI3K	PDGFRα	p-CREB(S133)
mTOR	PDGFRβ	SAPK/JNK
p-AKT(S473)	FGFR1	SRC
p-GSK3α/β(S21,S9)	ER	ErbB2
p-PKCα/β(T638,Y641)	Kit (c-)	HDAC2
PKCα	p38	Nkx2.2
PLCγ1	p-p38(T180,Y182)	olig1
p70S6RP	MET	olig2
4E-BP1	p21	SHH
Ras	PKD/PKCμ	
Actin	SGK	

Downregulated < 2-fold	
p53	STAT3
AKT	STAT3 (lower band)
RhoA	Fos
RhoB	p-Jun (S63)
SGK (lower band)	p-AMPKα (T172)

Upregulated
p-RB (S795)

Unchanged	
Tubulin	EGFR
GAPDH	GSK3β
p65/NFkβ	MEK

Table S3. Antigens

Antigen	Company	Catalog No.
GSK3beta	Cell Signaling	9315
p-GSK3alpha/beta (Ser21/9)	Cell Signaling	9327
P38alpha MAP Kinase	Cell Signaling	9217
p-p38 MAPK (T180/Y182)	Cell Signaling	9215
Met	Cell Signaling	3127
PI3K(p85)	Cell Signaling	4292
PKCalpha	Cell Signaling	2056
PKD/PKCmu	Cell Signaling	2052
p-PKCalpha/betaII (T638/641)	Cell Signaling	9375
Akt	Cell Signaling	9272
p-Akt (Ser473)	Cell Signaling	4058
Caveolin-1	Cell Signaling	3238
p-AMPKalpha (T172)	Cell Signaling	4188
p-Rb (S795)	Cell Signaling	9301
p65 NFkB (C22B4)	Cell Signaling	4764
CREB	Cell Signaling	9197
p-CREB (S133)	Cell Signaling	9198
ErbB2/Her2	Cell Signaling	2248
ErbB4	Cell Signaling	4795
MEK(1/2)	Cell Signaling	9126
Estrogen Receptor (ER)	Millipore	04-820
FGF Receptor 1	Cell Signaling	3472
c-Fos(Pan)	Santa Cruz	sc-52
HDAC2	Cell Signaling	2540
mTOR	Cell Signaling	2983
N-Ras	Santa Cruz	sc-31
Cyclin D1	Cell Signaling	2926
PDGF Receptor alpha	Cell Signaling	3174

PDGF Receptor Beta	Cell Signaling	3162
SGK	Cell Signaling	3272
PLCgamma1	Cell Signaling	2822
RhoA	Cell Signaling	2117
RhoB	Cell Signaling	2098
SAPK/JNK	Cell Signaling	9258
Src	Cell Signaling	2109
Stat3	Cell Signaling	9132
p-c-Jun II (S63)	Cell Signaling	9261
Kit (c-)	Cell Signaling	3392
p53	Cell Signaling	9282
IGF-I Receptor Beta	Cell Signaling	3027
p-Bad (S112)	Cell Signaling	9296
P21 Waf1/Cip1	Cell Signaling	2946
P70 S6 Kinase	Cell Signaling	2708
4E-BP1	Cell Signaling	9644
Nkx2.2	Developmental Iowa Hybridoma Bank	74.5A5
olig1	Chemicon	AB5991
olig2	Millipore	MABN50
EGFR	Millipore	06-847

Disease Models & Mechanisms • Supplementary information
